# The Gender Gap in Brazilian Entomology: an Analysis of the Academic Scenario

**DOI:** 10.1007/s13744-021-00918-7

**Published:** 2021-11-12

**Authors:** Juliana Hipólito, Leila Teruko Shirai, Rosana Halinski, Aline Sartori Guidolin, Nivia da Silva Dias Pini, Carmen Sílvia Soares Pires, Ranyse Barbosa Querino, Eliane Dias Quintela, Eliana Maria Gouveia Fontes

**Affiliations:** 1 Instituto de Biologia, Univ Federal da Bahia, Salvador, BA Brazil; 2 Instituto Nacional de Pesquisas da Amazônia, Manaus, AM Brazil; 3 Instituto de Biologia, Univ Estadual de Campinas, Campinas, SP Brazil; 4 Escola Politécnica, Pontifícia Univ Católica do Rio Grande do Sul, Porto Alegre, RS Brazil; 5 Escola Superior de Agricultura “Luiz de Queiroz”, Univ de São Paulo, Piracicaba, SP Brazil; 6 Embrapa Agroindústria Tropical, Fortaleza, CE Brazil; 7 Embrapa Recursos Genéticos e Biotecnologia, Brasília, DF Brazil; 8 Embrapa Cerrados, Planaltina, DF Brazil; 9 Embrapa Arroz e Feijão, Santo Antônio de Goiás, GO Brazil

**Keywords:** Insects, Scissor effect, Leaky pipeline, Sucupira, Lattes

## Abstract

Although women are about half of world’s population, they are underrepresented in many sectors including academia and the research scenario in general. Gender gap in Entomology has been pointed out in other publications; however, data for Brazil has never been demonstrated. Here we provide a diagnosis for the Brazilian Entomology scenario in order to contribute to propositions towards disentangling the gender gap in general. We analyzed scientometric data for Brazilian Entomology focusing on gender disparity and on personal perceptions related to the gender gap through an online questionnaire. We detected a pervasive gender bias in which the scissor-shaped curve is the most representative effect of it: women were the majority in lower degree stages but the minority in higher degree stages (permanent positions and positions of prestige and power). We also observed mentorship bias and discussed these results in light of intersectionality and the COVID-19 pandemic. Gender differences were perceived differently by the questionnaire respondents considering age, gender, and parenting. With this data and analyses, we have provided elements to stimulate and support change to a healthier and more equitable academic space.

**Supplementary Information:**

The online version contains supplementary material available at 10.1007/s13744-021-00918-7.

## Introduction

Women are about half of the world’s population and yet, in most societies, women are underrepresented in many sectors (Hryniewicz and Vianna 2018; World Economic Forum 2021). Careers and jobs such as administrators, engineers, politicians, and scientists are typically considered men-dominated, while occupations like teachers, nurses, and secretaries are considered feminine. These stereotypes manifest as early as 6 years old (Bian et al. 2017), creating, reinforcing, and perpetuating the gender gap (von Rumker 1978). The gender gap (revealed considering economic participation and opportunity, education level, health, survival, and political empowerment) is a global phenomenon, and it is of everyone’s benefit (Nielsen et al. 2017; Ferrari et al. 2018; Davies et al. 2021), though not of everyone’s interest, if addressed through matching scenario strategies. The COVID-19 pandemic aggravated gender inequalities, retrogressing the path to balance (World Economic Forum 2021). If the strategies to achieve equity were always needed, now they must get stronger.

Publications discussing the gender gap in academia have gained propulsion in the 2000s (e.g., Xu 2008), despite studies on the topic existing since the 1950s (see Weston et al. 1986; Widnall 1988; Rossiter 1993). The existence of an academic leaky pipeline, or scissor-shaped curve (i.e., the phenomenon in which the proportion of women in academia progressively decreases with advancing career stages) has been reported in nearly every study with data on gender proportions along the academic career, from undergraduate and graduate levels to employment and positions of power (Pell 1996; Howe-Walsh and Turnbull 2016; Davies et al. 2021). This mismatch is even higher due to the increase of people worldwide reaching higher education (Bradley 2000), followed by lower numbers of academic job positions in the past years (Taylor 2011; Yamada 2019). This scenario favors a spillover effect marked by the replication of internal biases of a poorly diversified hierarchical chain strongly marked by a male, white, and cisgender perspective (Diele-Viegas et al. 2021).

Men are more likely to contribute with other men (Araújo et al. 2017; Walker 2020) and credibility is more likely given to men, the “Matilda effect” by which those at the top are over-recognized, while others (like women) are suppressed or forgotten into extant or posthumous obscurity (Rossiter 1993; Nature editorial 2021). The limited recognition is aggravated with intersectionality, that is, women who also belong to other minority groups (mothers, LGBTQ + , Indigenous, Black, Asian, people with disabilities, among others) that lack credibility, opportunities, and live with bullying or racism/sexism/ableism/etc. by their peers (Feir et al. 1990; Abramson et al. 2013; Khan et al. 2019; Hipólito et al. 2020; Staniscuaski et al. 2020; Turney et al. 2020; Diele-Viegas et al. 2021). Female researchers during COVID-19 pandemic, for example, published less than male academics, and among them, Black women and mothers were the most negatively impacted (Staniscuaski et al. 2021). To dismantle this system that benefits privilege over diversity and inclusion, the first step is to examine the composition of research spaces and determine where representation is lacking (Chaudhury and Colla 2021; Davies et al. 2021).

The gender gap is everywhere but the amplitude of the bias can be context-dependent as, for instance, the perception of masculinity differs among scientific disciplines (Makarova et al. 2019). Some fields like Economics, Law, and Agronomy are considered man-disciplines, but even in fields that do not, on average, carry an obvious gender stereotype, such as Biological Sciences, sub-fields can be strongly marked by gender bias, like Entomology (Rockey and Jaworski 1987; Richmond and Whitney 1990; Feir et al. 1990; Abramson et al. 2013; Evangelista et al. 2020). Also, the historical context of social mixture or co-existence of ethnicities might influence gender disparity in societies today; for example, even considering the shared history of colonialism in the New World, it happened very differently between North and South America (Elsevier 2021).

Entomology, defined as the study of insects, is an important discipline since insects have direct importance in many human activities and are prevalent in every aspect of animal diversity, such as richness, abundance, biomass, and life forms (Wilson 1987). As insects are one of the most diverse clades on Earth, their study has multiple fields and facets performed by various disciplines, such as Agronomy, Biodiversity, Conservation, Ecology, Genetics, Public Health, and Zoology. Discussions in the academic literature concerning the gender gap in Entomology began to appear in 1980s from conference meetings of the world’s largest Entomological Society (Rockey and Jaworski 1987; Richmond and Whitney 1990); however, it was only in 2018 that scientometric data quantitatively showing the gender gap for professional Entomology in the USA appeared (Walker 2018), followed by other studies later on, in the same country (Walker 2020; Evangelista et al. 2020).

It is remarkable that, despite so many studies detecting gender disparity in the USA, it ranks in the 30th position of the Global Gender Gap Index (World Economic Forum 2021). We can only understand general mechanisms and venues of change if we consider challenges and opportunities faced by women scientists of other countries and contexts. Another continental country (the fifth largest on the planet), among the 10 most populous in the world (ca. 213 million people) (IBGE 2021), is Brazil. Brazil holds the largest rainforest and savanna in the world, indispensable biocultural diversity (Gorenflo et al. 2012), and many conservation hotspots (Myers et al. 2020), and is home to many clades, including insects, whose adaptive radiation happened in the Neotropics (Robbins and Opler 1997). Unlike its biodiversity, Brazil sits on the 93rd position of gender diversity (World Economic Forum 2021). Brazil has only 13.8% of Political Empowerment gender gap, and few women parliamentarians (15.2%) and ministers (10.5%), and only one woman was elected as president for 5 years considering an interval of 50 years. On the past years, Brazilian science has been facing progressive budget cuts, more severely in recent years (Oliveira et al. 2020; Tollefson 2020), which increase competition and intensify the lack of diversity by e.g. reinforcing the *Matilda effect* in science.

With that scenario in mind, diagnosing the Entomology scenario in Brazil with scientometric data might contribute to propositions towards disentangling the gender gap in academia generally. As far as we know, there is no such data for this country despite a healthy community discussing the academic gender gap in social networks. We thus aimed to analyze the (1) Brazilian Entomology academic scenario focusing on gender disparity, and, considering that numbers could not give us the entire gender scenario in Entomology, we expanded our analysis to (2) personal perceptions of researchers related to gender gap through an oriented online questionnaire. In a follow-up paper, we discuss the Brazilian Entomology scenario of publications and editorial policies.

## Material and methods

### Brazilian Entomology academic scenario

**Fig. 1:**
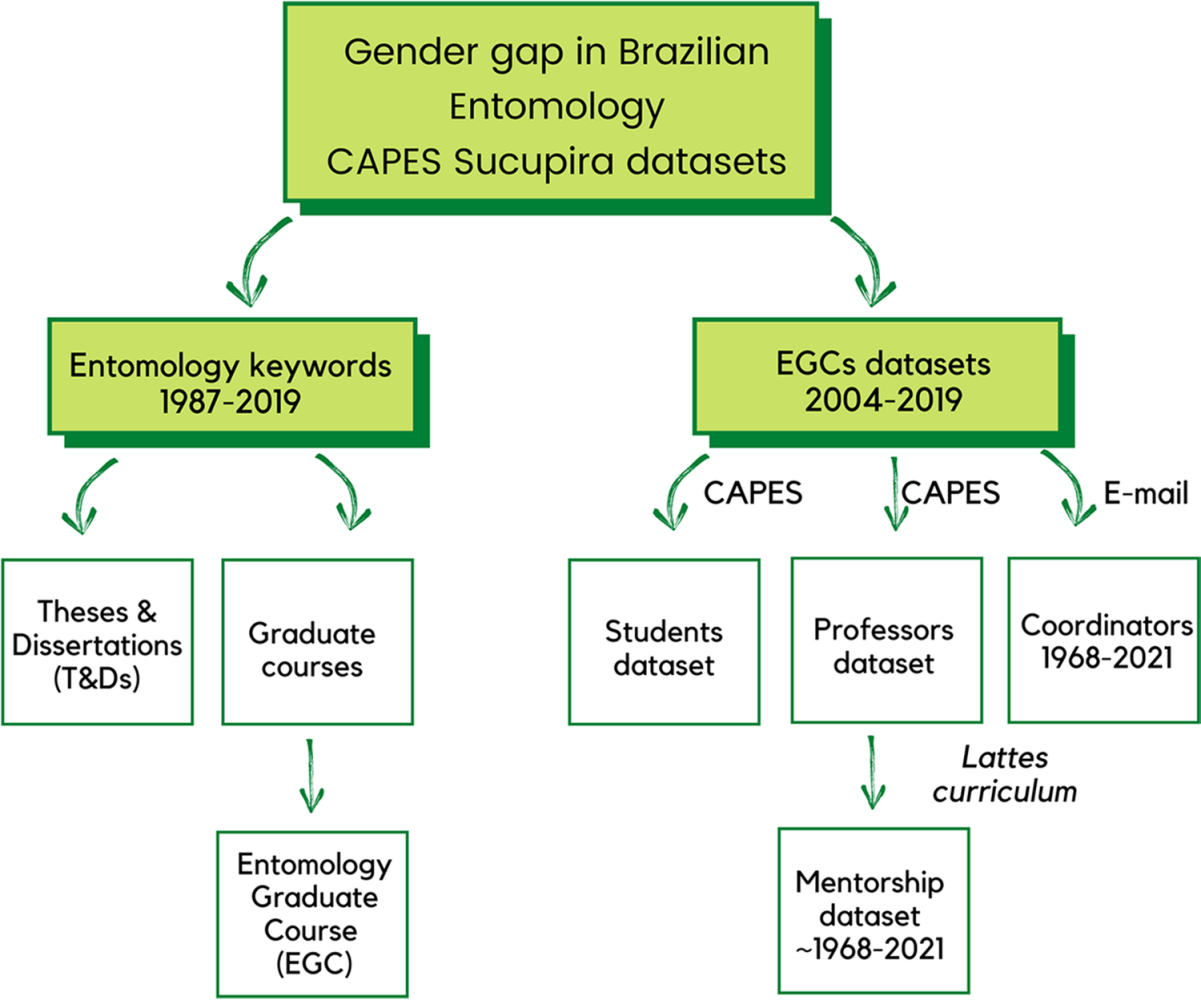
General scheme summarizing CAPES Sucupira datasets used in the present study of the gender gap in Brazilian Entomology. “Entomology keywords” (Fig. 2) and “Entomology graduate courses” (EGCs, Figs. 3 and 4) corresponded to different time intervals for the publicly available data that we had access to. Those two major groups of datasets were used to analyze other datasets (indicated by arrows, see more in Suppl. Mat. 1). In “EGCs datasets” the source of the information for that dataset is written alongside the arrow. For “Entomology keywords,” they all derive from CAPES

To provide the first diagnosis of the gender gap in Brazilian Entomology academic scenario, we used datasets (Fig. 1) from the publicly available *Coordenação de Aperfeiçoamento de Pessoal de Nível Superior* (CAPES) federal database, through the *Sucupira* platform (https://dadosabertos.capes.gov.br/dataset?organization=diretoria-de-avaliação, accessed Dec 12th, 2020). In this large amount of information, we found quite low rates of database error; however, data curation was mandatory (see Suppl. Mat. 1) to avoid, for example, double counts of the same person.

We manually assigned sex (binary: female or male) for researchers when information was not available in the CAPES database. We used given names to categorize sex since culturally in Brazil most first names are distinguishable. In neutral given names, if the person had multiple given names and one was neutral but not the other, we assigned sex by the other, like Muriel Fernanda as a female name, or Muriel Marcelo as a male. When given names were (all) neutral, we searched the person’s full name in the most complete and up to date repository of Brazilian students and researchers: the online database of the *Conselho Nacional de Desenvolvimento Científico e Tecnológico* (CNPq) *Lattes curriculum* (buscatextual.cnpq.br/buscatextual/busca.do?metodo = apresentar), looking for evidence to assign sex, such as “*Eu sou pesquisadora de abelhas*” (I am a bee [female] scientist).

As the *study of insects* is a complex and multidisciplinary field, we laid down in detail elsewhere the decisions for which keywords would represent it, as well as exploratory analyses and data curation (Suppl. Mat. 1). From now on, we treat the study of insects and Entomology indistinguishably, meaning that any person researching insects at any level for any number of years was contemplated here. Likewise, by Brazilian entomologist, we mean Brazilians who acted in the country but may today be abroad, or foreigners who study insects in Brazil.

We firstly gathered information from all Master Theses and Doctoral Dissertations (T&Ds) defended from 1987 to 2019 in every graduate course in the country recognized by CAPES. We then searched for keywords in the T&Ds titles (“Entomology keywords” in Fig. 1, we illustrated a word cloud, i.e., a visual representation of the most frequent words in resulting Entomology T&Ds titles, in Fig. S4), using additional columns (such as year, main area, institution; see Suppl. Mat. 1) for data curation, as well as for exploratory and analytical purposes. We then chose the 12 existing Entomology graduate courses (EGC, “EGC datasets” in Fig. 1) in Brazil (see why in Suppl. Mat. 1) to further explore student, post-docs and professors’ profiles, as well as to reveal patterns of mentorship, explained in the next section.

### Entomology graduate courses (EGCs)

We analyzed the 12 Brazilian ECGs (“EGC datasets” in Fig. 1) using (1) two datasets of the Sucupira platform, one concerning profiles of Masters and Doctoral students (hereon Sucupira students dataset), and another of professors profiles (hereon Sucupira professors dataset), filtering data of the 12 EGCs by their unique code (Table 1); (2) based on the Sucupira professors dataset, we counted the number of students and post-docs by sex of each EGC professor in their online *Lattes curriculum*, to which we will refer as EGC mentorship dataset; and (3) historical information of names and mandates of all EGC coordinators directly provided by the current one. The Sucupira students and professors datasets ranged from 2004 to 2019. In these two Sucupira datasets, the sex of the person was informed.

**Table 1:** Entomology graduate courses (EGC) codes according to CAPES, with their total number of theses and dissertations (T&Ds), their Brazilian region and state, and their institutional name and acronym

EGC code	Number of T&Ds	Region	State	Acronym	EGC university name
32002017016P0	524	South-East	MG	UFV	Universidade Federal de Viçosa
40001016005P5	487	South	PR	UFPR	Universidade Federal do Paraná
33002037001P7	475	South-East	SP	USP/ESALQ	Universidade de São Paulo/Escola Superior de Agricultura Luiz de Queiroz
33002029018P1	389	South-East	SP	USP/RP	Universidade de São Paulo/Ribeirão Preto
12002011004P6	353	North	AM	INPA	Instituto Nacional de Pesquisas da Amazônia
32004010007P8	313	South-East	MG	UFLA	Universidade Federal de Lavras
33004102037P9	306	South-East	SP	UNESP/JAB	Universidade Estadual Paulista Júlio de Mesquita Filho/Jaboticabal
51005018003P9	193	Mid-West	MS	UFGD	Universidade Federal da Grande Dourados
25003011017P3	151	North-East	PE	UFRPE	Universidade Federal Rural de Pernambuco
42003016046P1	28	South	RS	UFPEL	Universidade Federal de Pelotas
51001012014P5	18	Mid-West	MS	UFMS	Fundação Universidade Federal de Mato Grosso do Sul
33002010246P9	5	South-East	SP	USP/PH	Universidade de São Paulo/Public Health

We explored the Sucupira professor’s dataset with correlation analyses (Pearson product-moment correlation) with a two-tailed *t*-test for statistical significance. Based on this dataset, we produced the EGC mentorship dataset by manually counting the total number of undergraduate (or Bachelor degree, BD), Master (MSc), and Doctoral (PhD) students, and Postdoctoral (PD) researchers associated with each of them, using the professor’s online *Lattes curriculum* (accessed Jan 29th to Feb 8th 2021), with counts per sex inferred by the students or PDs given names, totaling 22.786 students and PDs (the same person can repeat if appearing at different levels). The time interval of EGC mentorships spanned all advisorships (the earliest defense year among the ten oldest professors’ dates from 1968) to ongoing, unfinished, ones in 2021. We did not consider co-advisorships, specialization courses, advanced training courses, and advisorships of “other nature.”

Furthermore, to explore the scissor-shaped curve in EGCs, we calculated the gender proportion in each career stage, also adding to the EGC mentorship dataset the level of each professor in the CNPq Productivity fellowship (*Bolsa de Produtividade CNPq*). Aside from a research allowance, this fellowship ranks professors in five levels (1A, 1B, 1C, 1D, and 2) which are seen as levels of prestige, sometimes considered in future grant raising. Although the Sucupira professor’s dataset provided this information, many professors had missing data for the most recent years, but the information was available in their *Lattes curriculum* so we checked all professors to standardize the source of the information (accessed Jul 10th to 11th 2021).

We used the Sucupira student dataset mainly to calculate time until defense or abandonment. We assessed significant differences between the time female and male names took to conclude or abandon their graduate degrees with the Wilcoxon rank sum non-parametric test in R software (R Core Team 2020) since, for most cases, the data was not normal. We reorganized the data to follow each student through time since the raw information would lead to double or incorrect counts of the same student.

### Personal perceptions of researchers related to the gender gap in Entomology

We reached out to Entomologists through social networks (WhatsApp, Instagram, Facebook, Twitter and podcast), e-mails and the SEB website (https://seb.org.br, *Sociedade Entomológica do Brasil*—Entomological Society of Brazil), which is the second largest entomological society in the world, after the Entomological Society of America), from February to April 2020, asking to voluntarily fill out an online questionnaire. The questionnaire (Suppl. Mat. 2 questionnaire), previously approved by the ethical research committee (*Comitê de Ética em Pesquisa*, *Certificado de Apresentação de Apreciação Ética*—CAAE: 42,387,021.8.0000.5336), contained 14 questions that considered personal (e.g., gender, color, age, children) as well as professional (e.g., workplace, job situation, field of knowledge) information and a couple of open questions related to personal perspectives about gender perception (Suppl. Mat. 2). Unlike the CAPES datasets where we only had information about sex, we asked participants their gender identity, understood here as coming from a person’s feeling and self-declared opinion, which may not correspond to the person’s physiology or designated sex at birth.

For this data, we calculated basic descriptive statistics such as percentage of distribution, mean and standard deviation of respondents, demographic data, and work-related variables. For the analysis of internal consistency, we performed Cronbach’s alpha test considering 0.7 or above as acceptable (Cronbach 1951). We performed normality tests to verify data distribution and chi-square (*χ*^2^) tests to relate sociodemographic variables with differences in perception about gender and the presence/absence of challenges and opportunities for different gender identities (see Suppl. Mat. 2), with *p* < 0.05 being considered significant.

## Results

### Brazilian Entomology academic scenario

**Fig. 2:**
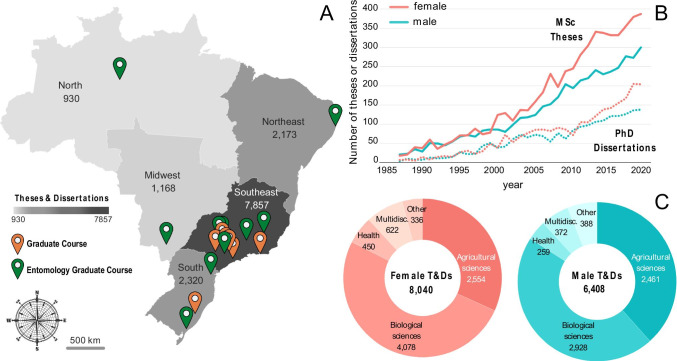
Entomology keywords results showing in **A** map of Brazilian regions showing the total number of theses and dissertations (T&Ds); darker areas highlight the higher number of T&Ds. The 20 graduate courses with highest number of Entomology T&Ds are shown in orange and, in green, those that are Entomology graduate courses (EGC). **B** Number of T&Ds per Master (MSc, solid line) or Doctorate (PhD, dashed line) levels per year. **C** Number of T&Ds proportionally to each main area, with “Multidisc.” corresponding to Multidisciplinary science, and “Other” to Exact sciences, Engineering, and Humanities

The Sucupira platform lists more than 1.2 million (1,235,795) Master Theses and Doctoral Dissertations (T&Ds) at 4,918 graduate courses in Brazil from 1987 to 2019. Among these, we found 14,448 Entomology T&Ds (1%) at 1,224 graduate courses (25%) using 75 keywords related to Entomology (see Suppl. Mat. 1)(Fig. 2). Most of Brazilian graduate courses with T&Ds related to the study of insects were in Brazilian Southeast region and corresponded to a third of all T&Ds (Fig. 2A).

Considering all graduate courses related to the study of insects, 11 of them were responsible for 25% of all T&Ds: nine of these being Entomology graduate courses (EGC, shown in green in Fig. 2A, see inset graph in Fig. S5), and two Zoology courses. Brazilian EGCs educate future entomologists with many dedicated disciplines concerning insects; however, they are biased towards Agricultural Sciences, which does not necessarily reflect the diversity of other graduate courses that train people in the study of insects. For example, among the 54 graduate courses that produced half of Entomology T&Ds (shown in purple in Fig, S5), only 13 had Agricultural Sciences as the main area, while 38 were in Biological Sciences (Multidisciplinary, Health, and Exact Sciences represented 1 graduate course each).

The number of Entomology T&Ds increased over time, with higher numbers for female students starting in the 2000s (Fig. 2B). The difference between T&Ds defended by male and female names is higher for MSc degrees than for PhD degrees (Fig. 2B).

Comparing the percentage of females and males on Entomology T&Ds, we found the same pattern for the preponderance of main areas between sexes, but with different percentages: Biological Sciences (51% female and 46% male), followed by Agricultural Sciences (32% female and 38% male), Health Sciences (6% female and 4% male), and Multidisciplinary Sciences (8% female and 6% male), with remaining main areas with much smaller contributions (Fig. 2C). From the 14,448 Entomology T&Ds, we found 12,021 (83%) in the main areas of Agricultural and Biological Sciences corresponding to, respectively, 3.8% (5,015 in 129,265) and 7.3% (7,006 in 95,060) of their total T&Ds.

### Entomology graduate courses (EGCs)

**Fig. 3:**
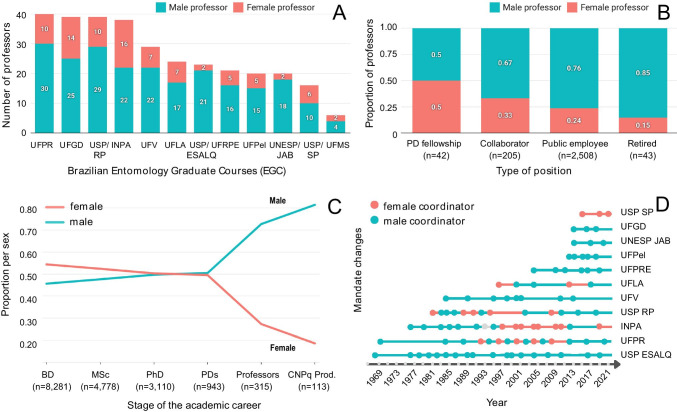
Leaky pipeline in Brazilian Entomology, represented by the Entomology graduate courses (EGCs). **A** Number of female and male professors in EGCs. **B** Proportion of professors in different job positions. The total number (n) per job position is given below each position. **C** Proportion of female and male students (Bachelor Degree “BD,” Master “MSc,” and Doctorate “PhD”) and postdoc (PDs) for each of the EGC professors, as well as their status in the CNPq Productivity fellowship (“CNPq Prod.”). **D** Changes in mandates of EGC coordinators, each dot corresponding to a different person from the coordinator of the previous mandate. The grey dot represents a mandate with missing data

Considering exclusively the 12 Brazilian Entomology graduate courses (EGCs), we found a large discrepancy between the number of female and male professors. From 2004 to 2019, we found 86 female and 229 male professors (Fig. 3A). Among those, the time in the institution also differed as we found male professors for longer periods on those institutions than female professors (63% from 1 to 5 years, 84% from 6 to 10 years, and 75% from 11 to 16 years, percentage of male professors). Equal proportions between male and female were only found at temporary positions (e.g., research grants or fellowships related to PD positions), decreasing the sex proportion in permanent positions (Fig. 3B).

Regarding professors’ age, we found that EGC professors were born from 1922 to 1988, and despite the cross generational profile and regional diversity (Fig. 2A), they have become doctors with little variation: 35 ± 5.63 years (average age ± standard deviation; between year of birth and year of PhD defense with *R* = 0.92, *T* = 14.660, *p* < 0.01) for female and 33 ± 3.9 years (*R* = 0.97, *T* = 29.541, *p* < 0.01) for male. Among these EGC professors, the first male doctor obtained his PhD in 1951 and the first female doctor, in 1976.

We did not have access to information for when they were hired, preventing us from analyzing the time after hiring relative to mentorship, that is, the number of student (BD, MSc, PhD) advisership and PD supervision. But looking at the sex proportion at all level (students, PDs, professors who advised or supervised them and their CNPq Productivity fellowship status, Fig. 3C), we found more female at earlier stages (BD and MSc), as seen in the total Entomology T&Ds (Fig. 2B). At the PhD and PD levels, proportions between female and male became similar. After this stage, however, there is an abrupt inversion in the proportion of male names, holding 73% of EGC professorships and 81% of CNPq Productivity fellowships (Fig. 3C). By looking into another position in the career, that of the coordination of EGC programs, the higher proportion of male EGC coordinators is even more striking (Fig. 3D).

There is a positive correlation of the number of T&Ds with the age of the EGC (*R* = 0.85, *T* = 5.103, *p* < 0.01; see Fig. S6), as well as with the total number of professors in each EGC (*R* = 0.63, *T* = 2.565, *p* = 0.03). The correlation is also positive and significant for male (*R* = 0.77, *T* = 3.816, *p* < 0.01) but not for female (*R* = 0.24, *T* = 0.782, *p* = 0.45).

**Fig. 4:**
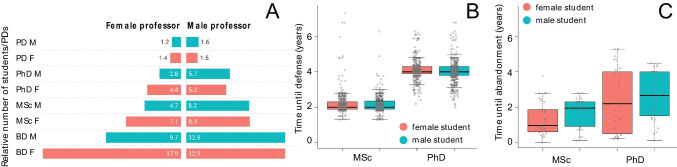
**A** Proportion of female (F) and male (M) students (Bachelor Degree “BD,” Master “MSc,” and Doctorate “PhD”) and postdoc (PDs) by male and female professors (indicated by colors) on the 12 Brazilian Entomology graduate courses (EGCs). **B** Boxplots with the individual values plotted demonstrating the difference of female and male students related to their time until defense per level and **C** as in **B**, for time until abandonment of the graduate course (years)

We also quantified how many female and male students and PDs were advised by female and male professors. Given the disparity of the absolute number of female and male professors, we analyzed numbers of students and PDs relative to the total of professors by sex. Female professors advised more female BDs, but all other mentorships (level*sex) had higher relative values for male professors, especially for male students (Fig. 4A). Additionally, we found some professors with particularly high numbers of students. Among the top 10% adviserships at each level (BD, MSc, PhD), we found six researchers (five of which are men) in the top 10% for two or more levels, responsible for 8% of all adviserships. Individually, each advised 96 to 318 students (we do not include PDs in adviserships). Advisors’ age in 2020 ranged from 46 to 76 years old. The proportion of their students was 64% male students and 36% female students.

We found 2,360 students in the Sucupira students dataset. The time MSc and PhD students took to obtain (Fig. 4B) or abandon (Fig. 4C) the academic degrees did not differ between sex, except for defended PhDs (W = 178,410, *p* = 0.02), but the difference is minimal (Fig. 4B, median female = 4.09 years and male = 4.02 years; 0.01 year corresponds to 3.65 days). We found tendencies (Fig. 4B) evidencing that female MSc students took longer to defend (75–100% quantile, female 2.3 to 6.8 years and male 2.4 to 4.9 years, *W* = 10,626, *p* < 0.01); and some male PhD students obtained their degree in less time than female students (0–25% quantile, female 2.4 to 3.9 years and male 2.0 to 3.8 years, *W* = 525, *p* < 0.01).

### Personal perceptions of researchers related to the gender gap in Entomology

We had 1,253 respondents in our questionnaire from all Brazilian states, with 59.9% women, 38.3% men, 0.3% non-binary, and 1.5% of another gender identity (Table 2). The average age of respondents was 35 ± 9.3 years. Most respondents had a PhD (45.8%) or a PhD in progress (18.2%), with the majority working in academia (71.4%).

**Table 2:** Summary of respondents from the questionnaire related to the personal perceptions of researchers related to the gender gap in Entomology

Characteristics	*N*	%
Gender identity		
Men cis	469	38.1
Men transexual/transgender	3	0.2
Woman cis	732	59.5
Woman transexual/transgender	5	0.4
Non binary	4	0.3
Other	18	1.5
Self-declared color		
Asian	11	0.9
White	772	61.8
Indigenous	4	0.3
Black	89	7.1
Brown	341	27.3
Others	32	2.6
Age		
19–29	445	36.4
30–39	411	33.6
40–49	187	15.3
50–59	122	10
60–69	41	3.4
70–83	16	1.3
Deficiency		
No	1214	97.2
Yes, hearing impaired person	3	0.2
Yes, physically disabled person	5	0.4
Yes, visually impaired person	16	1.3
Others	11	0.9
Parenting		
Yes	393	31.4
No	860	68.6
Number of children		
1	182	46.7
2	159	40.8
3	40	10.3
4	9	2.3
Maximum Education		
Technical education	3	0.2
Bachelor degree in progress	143	11.4
Bachelor degree	72	5.8
MSc in progress	159	12.7
MSc	73	5.8
PhD in progress	228	18.2
PhD	572	45.8
Year of conclusion of Maximum Education		
Before 1980	8	0.7
1981–1990	16	1.4
1991–2000	75	6.8
2001–2010	208	18.8
2011–2020	710	64.0
2021	92	8.3
Federation Unit of your main workplace		
Acre	4	0.3
Alagoas	23	1.9
Amapá	11	0.9
Amazonas	69	5.6
Bahia	54	4.3
Ceará	25	2.0
Distrito Federal	45	3.6
Espírito Santo	23	1.9
Goiás	28	2.3
Maranhão	25	2.0
Mato Grosso	34	2.7
Mato Grosso do Sul	25	2.0
Minas Gerais	181	14.6
Pará	62	5.0
Paraíba	20	1.6
Paraná	98	7.9
Pernambuco	46	3.7
Piauí	24	1.9
Rio de Janeiro	60	4.8
Rio Grande do Norte	19	1.5
Rio Grande do Sul	91	7.3
Rondônia	5	0.4
Roraima	5	0.4
Santa Catarina	26	2.1
São Paulo	217	17.5
Sergipe	12	1.0
Tocantins	10	0.8
Number of job positions		
Unemployed	75	6.1
1	1107	89.3
2	52	4.2
3 or 4	5	0.4
Years have you worked in this location		
0–1	115	9.5
1–5	583	48.1
6–10	247	20.4
11–15	118	9.7
16–20	48	4.0
21–25	27	2.2
26–30	22	1.8
31–35	24	2.0
36–40	12	1.0
41–45	10	0.8
Mais de 45	5	0.4
Work in any innovation sector		
Yes	296	23.8
No	948	76.2
Have LinkedIn		
Yes	700	56.1
No	548	43.9
Work in the academic area		
Yes	893	71.4
No	357	28.6
Work in scientific publishing (e.g., editorial boards, reviewer)		
Yes	232	26.0
No	660	74.0
Challenges and opportunities are the same for professionals regardless of gender		
Yes	393	31.7
No	845	68.3

When we asked whether the person agreed or not that challenges and opportunities in the labor market are the same for professionals regardless of gender, most respondents (68.3%) considered that it was not the same (Table 2). We found significant differences in this perception by age group (*χ*^2^ = 75.538, df = 5, *p* < 0.001); gender (*χ*^2^ = 37.728, df = 3, *p* < 0.001); parenting (having or not children) (*χ*^2^ = 33.814, df = 1, *p* < 0.001); academic degree (*χ*^2^ = 15.393, df = 6, *p* = 0.017); and region of the country (*χ*^2^ = 11.346, df = 4, *p* = 0.023) (Suppl. Mat. 2). We did not find, however, significant association between respondent’s perception with the self-declared gender (men or women cis, men or women trans, non-binary; *χ*^2^ = 5.312, df = 5, *p* = 0.257), though it can be due to the low sample sizes for some gender categories.

We obtained a divided opinion when we asked about the perception of entomologists on gender-related differences in their work expertise: 49.7% noticed differences and 48.7% not, with 1.6% unable to give an opinion. Despite that, we found significant differences when analyzing this perception by age (*χ*^2^ = 27.546, df = 10, *p* = 0.02); sex (*χ*^2^ = 68.049, df = 6, *p* < 0.001); gender (*χ*^2^ = 25.222, df = 8, *p* = 0.001); self-declared color/race (*χ*^2^ = 23.914, df = 10, *p* = 0.008); and region of the country (*χ*^2^ = 28.921, df = 8, *p* < 0.001) (Suppl. Mat. 2). Here, 100% of non-binary people reported to have experienced gender disparities, as well as 59% of women, but only 37% of men. We also observed that after 2011, more people in the highest academic degrees reported gender differences in their work expertise, which was not observed before this year.

## Discussion

The Brazilian Entomology academic scenario reflected a pervasive gender gap (Figs. 2, 3, and 4) in which the leaky pipeline is the most representative effect of it (Fig. 3C). Although women are the majority at Bachelor and Master degrees (Figs. 2B and 3C), they do not occupy equivalent levels at permanent job positions and positions of power and prestige (Fig. 3). Some women do get hired and reach positions of power, but most women Entomologists are progressively abandoning, quitting, or being removed from the academic pipeline. The scissor-shaped curve has been found in several studies, like in the top 15 universities of the world (Khan et al. 2019), in ca. 9 million Brazilians in science and technology (Areas et al. 2020), or the Entomology scenario in the USA (Walker 2018), where men were overrepresented in permanent positions in academia and in the federal government, with better salaries. Some authors suggest that this pattern is related to “personal” (we would rather call it structural) reasons restricted to women, as they tend to drop out the competition or delay their careers for childbearing (Ceci et al. 2009; Adamo 2013) or to follow a partner to a job (Martinez et al. 2007; Wolfinger et al. 2008; Ceci et al. 2009).

However, the causes and consequences of gender inequality worldwide are by far more complex (Elsevier 2021). Underdeveloped countries for example tend to exacerbate favoritism towards males (Jayachandran 2015), which can come from more competition over less resources. Also, in less economically developed countries, where there is frequently less affirmative action or public policies towards diversity, the gender gap in different fields of higher education can be actually weaker (Bradley 2000). Despite appearing counter-intuitive, that might happen because less people reach higher education in these countries, and only the elite, the wealthiest or privileged boys and girls, are able to enroll universities (Bradley 2000). Regionality and other historical context-dependencies should be taken into consideration to understand global gender challenges. The first step is with data, and we hope to have contributed to that, as well as with the following discussion.

On Entomology (insect studies) theses and dissertations (T&Ds), we found slight tendencies of Agronomy T&Ds towards male students, and Health Sciences T&Ds towards female students (Fig. 2C). Historically, Agronomy is a field typically associated with male stereotypes and strongly dominated by men (Bradley 2000). We also saw these biases reflected on personal perceptions, following traditional stereotypes and global patterns among disciplines (e.g., Bradley 2000). Moreover, most T&Ds were at the Southeast region of Brazil (Fig. 2A), partly reflecting its biodiversity, but more so the higher presence of universities, access routes, and money in the country (Oliveira et al. 2016). The Southeast is where exploration is oldest, perpetuating the Brazilian patriarchal overexploitation heritage, but it also has a strong academic system that attracts better researchers, pays better, engages more with the public, and ascertains itself (Ferrari et al. 2018). Many of these universities were created to support regional economic development, including some of the Entomology graduate courses (EGCs).

At Brazilian EGCs, the mentorship patterns suggested there can be gender mentorship bias (Fig. 4A): we found female professors advising more female students at early stages, which require more attention for less academic recognition (Ferrari et al. 2018). At the other academic stages, when more publications and future peers can be involved, male professors advise more, especially male students. Another study quantified students and PDs between male and female researchers at the Brazilian Academy of Sciences (Ferrari et al. 2018) and found higher numbers for female professors’ advisorships at almost all academic stages, including Agricultural Sciences. We are unsure about comparing it with our results since Ferrari and collaborators (2018) did not partition the sex of students. If more female students would similarly be present at earlier stages in their dataset, the mentorship pattern could show a different picture, with structured gender bias as in our results.

The number of T&Ds at EGCs expectedly correlates with how long they exist, and with the number of professors. Interestingly, the correlation remained high (0.77), positive, and significant with the number of male, but not female professors. This can be partly explained with female professors taking slightly longer to become doctors, together with the fact that the earliest female professor got her PhD in 1976 (earliest male in 1951), which could comparatively delay when they are hired as professors, but we believe there may be extra factors to consider.

Academia today is frequently a path carved by those with Doctoral degrees despite many issues in following this avenue, including anxiety or depression due to pressures and uncertainty on job opportunities (Taylor 2011; Woolston 2017; Yamada 2019) that are more probable and heftier for minorities (Evans et al. 2018) (see also intersectionality references in the “Introduction”). The absolute number of advisees that some professors had (like 318 students in 40 years — counting from the year of PhD defense, and not job position) suggested a low probability that the professor individually advised them, probably reflecting pyramid lab cultures (where PDs advise PhDs, who advise MScs, who advise BDs — usually without recognition), shared responsibilities with (junior) co-advisors, or no advisorship at all.

Some metrics, as the prestigious CNPq Productivity fellowship, might reinforce the need of quantity over quality as for example, this metric ranks professors based on the number, and not quality, of adviserships (as has mistakenly been confused in the retracted paper of AlShebli et al. 2020). Students learn from their advisors how to do science, and any science that is presented to them (like publish or perish, salami science, and the Matilda effect) will serve, at least for a while, as their model of conduct (Montgomery 2020). Also, advisors who do not guide students to follow their path, stimulating the student gain of intellectual autonomy, can narrow some possibilities that might benefit science, society, and even the professors themselves (Montgomery 2020): 28.6% of respondents in our questionnaire were not from academia, and 23.8% act on the private sector (Table 2). Increasing the bilateral exchange of knowledge has the potential to increase efficiency and productivity of both sectors and might reduce social and environmental externalities in the process.

The significant differences between male and female time of defense could also be related with the gender bias in mentorship. Some PhD male students in the lowest quantile became doctors faster than female students (Fig. 4B). Some female MSc students in the highest quantile needed more time to finish their theses (Fig. 4B). In both cases, female students took longer because they might find challenges (like maternity or harassment) and are not supported, or because they are simply not treated with the same enthusiasm or benefits as male students (Bagilhole 1993). Female students tend to enroll STEM degrees when they have a female rather male science advisor (Canaan and Mouganie 2021). We found some support for these suggestions in the preponderance of male students by male advisors at stages where they can more likely contribute as peers. In the questionnaire, there may be some hint towards this discussion since almost 75% of women agreed with gender inequalities in challenges and opportunities (Suppl. Mat. 2). The time MSc and PhD students took to abandon their degrees did not differ between sexes (Fig. 4C), but that does not say anything about the reasons for quitting academia. A further investigation dedicated to this topic is much needed in Brazil, especially if extended to other gender identities and intersectionality with, for example, ethnicity.

The leakiest moment of the academic pipeline is after the PhD and PD phase. The number of MSc and PhD CAPES fellowships increased since the 2000s (Fig. S7), perhaps reflecting in the higher number of students, found especially for female students (Fig. 2B). More graduate fellowships were not, however, followed by a proportional number of job positions (Taylor 2011). The expectation that increasing the level of higher education of women would lead to their empowerment does not hold if there are no jobs for them (Bradley 2000) or if available jobs are controlled by a system that silently expels or attempts to transform them into the dominant class (Feir et al. 1990; Bradley 2000).

The gender gap in Entomology is persistent over time, and these biases can affect job competition (Walker 2018). The lower number of female Entomologists means a lower number of female mentors to graduate students which, in turn, generates a looping effect where young women do not want to follow the career path, as they do not visualize themselves in it (Shen 2013). The same rationale applies to discourses of women inferiority, which is a powerful discourse (because people believe in it, c.f. women with impostor’s syndrome) but not a judicious one since, for example, women productivity is the same as men (Leta 2014; Huang et al. 2020) which we also found for publication and impact of those publications in Brazilian Entomology, reported in the follow-up study. These issues are not exclusively feminine, as we generally lack diversity in academia in a broader sense, with other minority groups being even less represented in an environment led by a white male cis hetero model (Turney et al. 2020; Diele-Viegas et al. 2021).

In Brazil, despite having one of the largest biocultural diversities in the world (Gorenflo et al. 2012) and most of the population self-declaring as non-white people (IBGE 2019), we received in our questionnaire more responses from white declared respondents. Interestingly, in asking about the perception of gender differences in their work expertise, most black and brown entomologists responded there is no such difference (Suppl. Mat. 2), despite disagreeing that challenges and opportunities are similar among gender identities (Suppl. Mat. 2).

In the questionnaire, we had access to sex and gender, but few non-white people responded, even less considering the interaction with ethnicity, and we would not assume this reflected a real lack of gender and intersectional diversity in Brazilian Entomology. Had we the access to gender and ethnicity in the Sucupira datasets, a more complex and intersectional picture could be revealed, like a double scissor-shaped curve where white men led senior positions, followed by white women, then non-white men, with a smaller percentage in the workforce at senior positions (Khan et al. 2019). We could not analyze the interaction of gender*race because there is no such data available and one important place to start the change would be at the platforms we used, CAPES and CNPq *Lattes curriculum*. These databases are outstanding in the quantity and quality of data, forming the basis of scientometric data in the country (e.g., Leta 2014). But unrecognizing gender and ethnic diversity (self-declared color has only recently been included in the *Lattes curriculum*) is a form of exclusion, either because the individual experiences the lack of choice to self-declare as he/she pleases, but also by not allowing disparities to be revealed with information.

The power of knowledge can be intense and irreversible. Academic gender bias papers are growing (e.g., Xu 2008), and discussions in several spaces, including social networks, allow unprivileged people to awake and understand they can speak and do not need to be treated differently than others. Equally important, privileged people might also recognize they have privileges, which is the first step to understand that others do not share them (see, for example, the opposing perceptions for privileged *versus* unprivileged people about gender equality in challenges and opportunities in Suppl. Mat. 2). People can certainly understand the gender gap better when they experience it, like a small change in perception about gender differences in their work expertise only at the PhD level, or denial of it for people over 40 years, who might already have gotten their jobs (Suppl. Mat. 2). Despite anecdotal due to low sample sizes, we found it interesting that people over 60 perceived challenges and opportunities as being the same among gender identities (Suppl. Mat. 2), likely because this generation of Brazilian entomologists did not go through the highly competitive hiring system of today.

Another important venue of change is affirmative action. We found 235 male professors in EGCs compared to 87 female professors. If we would increase the number of women affiliated to EGCs to 10 each year, while maintaining the number of men, we would take at least 15 years to have the same number of men and women. However, we know that this projective scenario is impossible due to investment cuts in Brazilian STEM’s scenario (Oliveira et al. 2020) that affect more intersectional women (Staniscuaski et al. 2021), and probably there has never been a scenario where only the number of women was allowed to increase — realistically, good case scenarios estimates that e.g. by 2080 we could achieve diversity (Gibbs et al. 2016). We are not advocating in favor of including women into the job board in spite of men. Instead, we advocate that opportunities, metrics, and measures that favor equity are needed if we are to favor better and more efficient science (Nielsen et al. 2017; Ferrari et al. 2018; Davies et al. 2021), which also has the positive externality to more humanitarian practices (Evans et al. 2018; Evangelista et al. 2020).

In 2021, the CNPq accepted the request of Parents in Science movement to include maternity leave in the *Lattes curriculum*. The maternity leave has already been considered in a handful of hiring processes, grants, and some areas within the CNPq Productivity fellowship. This is a great advance, as has the potential to equilibrate the time that mother’s stay out of academia (maternity leave); however, the road to achieve equity is longer than that. The COVID-19 pandemic deepened the gender gap, and there has not been short-, medium-, and long-term propositions done so far (Hipólito et al. 2020). Academia could notice, however, that society is changing, favoring diversity and inclusion over privilege, and perhaps leading people or institutions that already serve as models would achieve stronger impact and gain higher benefits if they take affirmative action before others (see Khan et al. 2019).

Because leading institutions are dominated by white cis men, it would be relevant to bring and inspire change by carving new paths with — and not for — minorities (Feir et al. 1990; Abramson et al. 2013), possibly understanding a breakthrough idea that science can be done without superiority. Solutions to achieve equity might not be easy to be implemented due to, for example, political, administrative, and cultural barriers, yet they can include (but are not limited to) new evaluation metrics, inclusive policies, supportive working environments, and promotion of the inclusion and permanence of underrepresented groups (Diele-Viegas et al. 2021). Changing evaluation metrics is a widely discussed topic (e.g., Reece and Hardy 2017), and evidences for positive benefits for doing so are numerous. Academia and work environments in a broader sense should also consider flexible family care and institutional reports for gender equality, as well as psychological and cultural strategies (further details on Smith et al. 2015).

It is always worth acknowledging that there are stereotypes associating scientists (Miller et al. 2015; Carli et al. 2016) or leaders (McCright and Dunlap 2013) to white male, but these are just biases and biases can change (Raymond 2013). Also, and perhaps more importantly, if we as students and researchers would unite to show society the value of science in its many forms, done by a diversity of people guided by equitable principles, maybe we could reach a better status of a socially and financially recognized job, and our disputes would no longer be among ourselves, but with the real challenges out there.

## Supplementary Information

Below is the link to the electronic supplementary material.

Supplementary file1 (PDF 314 KB)

Supplementary file2 (PDF 87 KB)
