# Supplementary material for: The Gender Gap in Brazilian Entomology: an Analysis of the Academic Scenario
Source: Neotrop Entomol. 2021 Nov 12;50(6):859–72. doi: 10.1007/s13744-021-00918-7 (PMC8587496; doi:10.1007/s13744-021-00918-7)
Supplement: Supplementary file 1 — Supplementary file1 (PDF 314 KB) [file 13744_2021_918_MOESM1_ESM.pdf]

The gender gap in Brazilian Entomology: an analysis of the academic scenario

Supplementary Material: detailed Material and Methods

Brazil is the fifth largest country in the world, with more than 212.5 million people (IBGE 2020). Any study aiming to recover its historical patterns and to diagnose its populational trends quickly reaches continental proportions, which is amplified when the topic is inherently rich, like "Entomology". Entomology field, defined as the study of insects, not only encompasses one of the most diverse clades on Earth, but the study itself also has multiple facets. Entomology ranges from the public to the private sector, from academy to non-scientific activities, from basic to applied sciences. Even within academia, Entomology is done in a wide array of disciplines, such as Agronomy, Biodiversity and Conservation, Public Health, and Zoology. As such, finding the best set of keywords to represent Brazilian Entomology required exploratory analyses, detailed here. We also present in this Supplementary Material the reasoning for choosing only the twelve Entomology graduate courses (EGC), as opposed to other graduate courses (like Ecology, Zoology, etc.) that also study insects, for detailed analyses of EGCs, supervisors and students, and other questions of the main text.

We explored keywords in the realm of Master theses and PhD dissertations (T&Ds) defended from 1987 to 2019, based on open data of the *Coordenação de Aperfeiçoamento de Pessoal de Nível Superior* (CAPES) federal database. CAPES is a public foundation created in 1951, responsible for evaluating graduate courses in Brazil, financing research, promoting collaboration, and making available scientific data (https://uab.capes.gov.br/historia-e-missao, accessed Feb 23rd 2021).

CAPES catalogued more than a million graduates (1,235,795), at 4.918 graduate courses in the Sucupira platform (https://dadosabertos.capes.gov.br/dataset?organization=diretoria-de-avaliação, accessed Dec 12th 2020), in the period of 1987 to 2019. Although T&Ds are only one aspect of Entomology, we reckoned it would be a fair representation of keyword use in other platforms, like the lattes platform. Nonetheless, we kept our criteria flexible as to include any keyword that brought enough (1%) results exclusively, that is, not captured by any other keyword; but not if it brought considerable noise, that is, most studies unrelated to Entomology, as with some common names.

Our criteria and decisions were based on numbers. We made our searches in Portuguese, thus many criteria are specific to our language, but here we translate keywords to English (shown in brackets). The CAPES Sucupira platform provides data in Microsoft Excel files, and we explored the data in this software using the SEARCH formula, thus some of the criteria or search terms are also specific to this software. We explored the following columns with dynamic tables: year, Graduate course code, Graduate course name, Brazilian state, Institution Name, Main area (*Grande área*) code, Main area name, Discipline (*Área Conhecimento*) code, Discipline name, Author, Title, and Level (MSc or PhD). "Main area" relates to large divisions between Biological, Exact, and Human Sciences, but also within, like Biological Sciences main divisions of Agronomy, Biology, and Medicine. "Discipline" relates to more specialized fragmentations of Main areas.

In sum, our decisions were to: 1) search for all insect orders and some genera (*c.f.* Decision 2), but not other taxonomic levels, like family; 2) include genera of widely used and well-known model organism, disease vectors, and pests, like *Apis*, *Aedes*, *Spodoptera*; 3) include common names; 4) use radicals in specific cases like entom* for Entomology, entomofauna, etc.; 5) use scientific names in Latin in their full spelling; and 6) use Portuguese words in their correct spelling, including accentuation, hyphenation, or other Latin orthography like cedilla (ç). Below we detail each of these decisions, also reporting the steps taken for data curation.

We searched for keywords in the Title column – only 61 titles were blank in the total 1,235,795 T&Ds. We considered master and professional master's degrees the same level (MSc). The SEARCH formula in Excel is case insensitive and it finds the search term in any part of the given cell. This formula returns a value, the place of the first keyword letter in the sentence (*i.e.*, the title), which is meaningless. We replaced this number for 1, to use it for some calculations like the number of exclusive T&Ds of a given keyword. As we based many of our decisions on this number, we calculated it by: filtering out the T&Ds caught by insect orders and generic keywords (entom*, inset*, insect*) and summing the search results (substituted for 1) in the column of each keyword.

Decision 1: search for all insect orders and some genera (*c.f.* Decision 2), but not other taxonomic levels, like family

We searched for some insect families as a keyword and asked whether they are also captured by keywords of insect orders. For example, Pentatomidae had 298 results and, among these, 274 (92%) were also caught by "Hemiptera" or "Heteroptera." Among the remaining 24 titles, only 5 (2%) would not be recovered by insect orders and generic keywords. This example repeated itself enough times to decide that the taxonomic level of family does not bring enough exclusive results, being contemplated by insect orders; also not justifying the effort to explore which would be the best insect families as keywords. If insect orders are already so numerous, exploring insect families as keywords would be a much deeper maze.

The decision not to include insect families is, however, alleviated by the decision to search for every insect order, even those that yield 0 results (*i.e.*, were not in the title of any T&Ds in the Sucupira platform between 1987 and 2019), because this is relevant information for understanding which orders are not, or under, studied at graduate levels.

The list of insect orders prioritized natural groups based on recent phylogenetic hypotheses, but also orders that were once monophyletic or once an order because they were valid and, as such, widely used in the past. For example, fleas were once order Siphonaptera, today an infraorder, and now they belong to Mecoptera (Tihelka et al. 2020): we included both names as insect "order." We evaluated some phylogenetic hypotheses (Kristensen 1981, Kjer et al. 2006, Trautwein et al. 2012, Misof et al. 2014, Beutel et al. 2017, Chesters 2019) and selected two based on the representativeness of monophyletic names valid in the recent (Beutel et al. 2017) and past (Kristensen 1981) state of the art evaluations.

We also searched for orders in a High school (Amabis & Martho 1990) and undergraduate (Storer & Usinger 1979, Brusca & Brusca 2003) Biology textbooks purposefully outdated and, due to really low numbers of T&Ds exclusively caught by these keywords (shown in

parentheses), we excluded them from further consideration: Anoplura (1), Corrodentia (0), Mallophaga (1), Phasmatoptera (0), Thysanura (0).

Decision 2: include genera of widely used and well-known model organism, disease vectors, and pests, like *Apis*, *Aedes*, *Spodoptera*

Brazilian agricultural research is frequently prominent in international academic ranks (Glänzel *et al*. 2006, Leta *et al*. 2013), as well as in tropical diseases research (*e.g.* Zyoud 2016, González-Alcaide *et al*. 2018). Additionally, insect model organisms like *Drosophila* are widely studied everywhere, and here, the presence of A. Dreyfus and T. Dobzhansky inaugurated the population genetics program in the 40's (Glick 2008), establishing a productive fruit fly community until today. As tracing the history of specific genera is not the scope of this study, we composed a list of important genera to primarily explore what could be the contribution of entomological studies that do not explicitly write the insect order in the titles of their T&Ds. The insect genera we explored can be classified in three groups:

a) pests: based on the federal decree *Portaria 112 de 08 de outubro de 2018* (available at https://www.in.gov.br/materia/-/asset_publisher/Kujrw0TZC2Mb/content/id/45174395/do1-2018-10-15-portaria-n-112-de-8-de-outubro-de-2018-45174182, accessed Dec 27th 2020): *Helicoverpa*, *Chrysodeixis*, *Heliothis*, *Anticarsia*, *Spodoptera* (Lepidoptera); *Bemisia*, *Ceratitis*, *Anastrepha*, *Bactrocera* (Diptera); *Anthonomus* (Coleoptera); *Euschistus*, *Dichelops*, and *Diaphorina* (Hemiptera);

b) disease vectors: dengue/Zika/chikungunya fever (*Aedes*), malaria (*Haemagogus*, *Sabethes*, *Aedes*), yellow fever (*Anopheles*), Chagas disease (*Triatoma*), leishmaniasis (*Lutzomyia*), and elephantiasis (*Culex*); and

c) model organisms: *Apis*, *Atta*, *Bombyx*, *Drosophila*, *Gryllus*, and *Tribolium*, also adding *Trichogramma* (Hymenoptera) that is widely studied in Brazil as a biological control agent.

Three genera appeared with substantial number of T&Ds exclusively caught by these keywords (**Fig. S1**): *Aedes* (644), *Apis* (569), and *Drosophila* (455). Particularly for *Apis* and *Drosophila*, the percentage of T&Ds that only used the genus name in the title was quite high, respectively, 80% and 86% (**Fig. S1**). That is, we would only catch 20 and 14% of bee and fruit fly research if we do not include the genus. This result convinced us for the need to include insect genera as keywords, despite some data cleaning being necessary (detailed in the next section).

We are aware this decision can overrepresent studies of hand-picked genera. For example, the pest genera we chose sum to a maximum of 20 species, which represent 0.018-0.025% of the Brazilian insect diversity (estimated 80,750-109,250 species, Lewinsohn & Prado 2005; sometimes estimated to 400k species *e.g.* Rafael *et al*. 2012) or 0.002% of the world diversity (estimated 950,000 species, *op. cit.*). Even though most people know insects as harm or pest (Barua et al. 2012), and despite the fact that this bias reflects in the science we make, the percentage of harmful and pest species is negligible close to the real diversity and importance of insects. However, not including the chosen genera would be a worse trade off. Nonetheless, we hold some confidence that most entomological studies still explicitly write the order in the title,

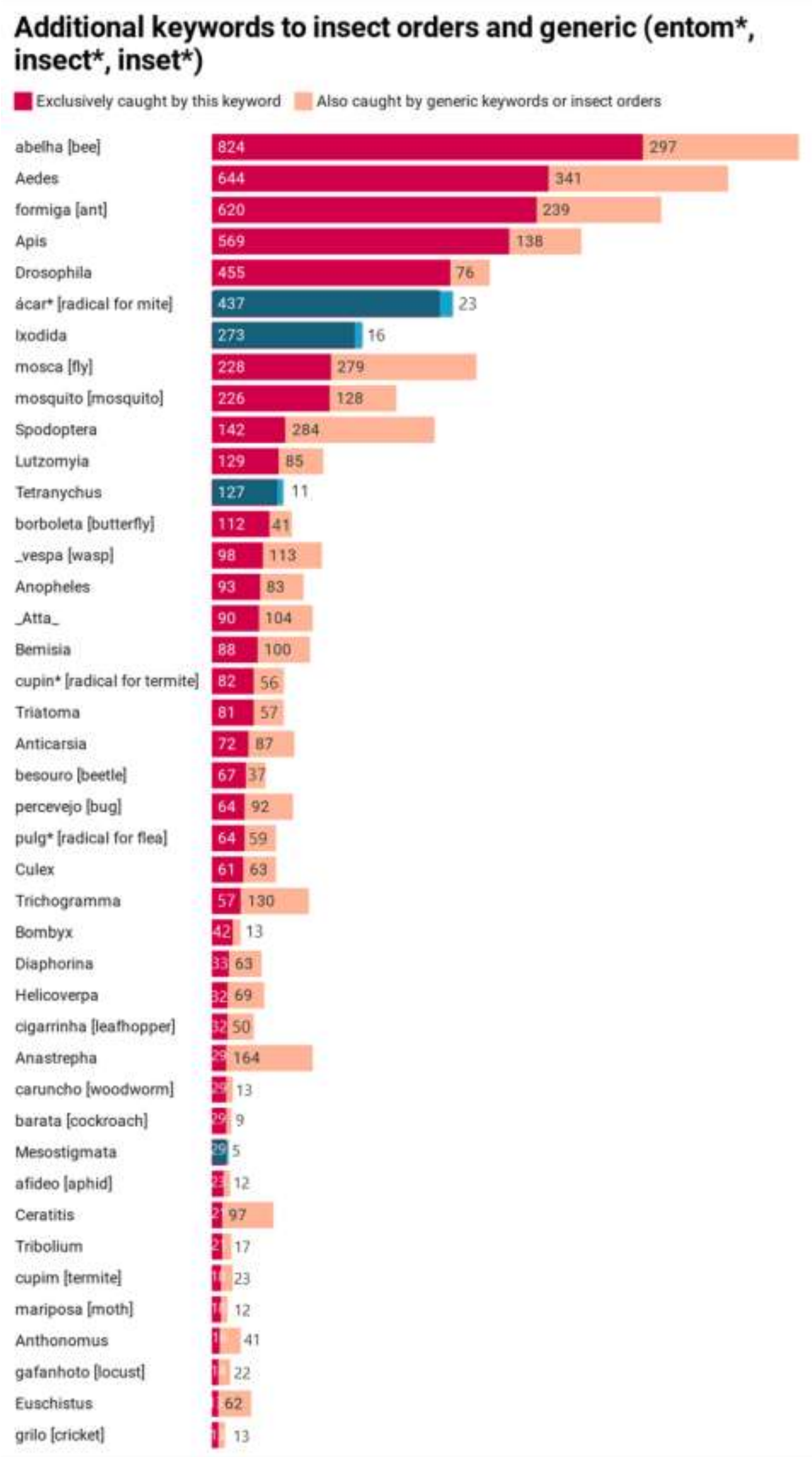

Figure S1: Number of T&Ds caught by additional keywords to insect orders and generic keywords. The number is shown as exclusively caught (*i.e.*, did not appear within results of orders and generic keywords, in dark pink) or not (light pink). Keywords are in Portuguese but were translated here in English (in brackets). * indicates radicals, underline indicates a space and, in blue, keywords related to mites (see last section).

or use generic words like “entomological” or “insect,” but diversifying the type of keywords is another important strategy, which led us to the common name keyword (Decision 3).

As in decision 1, we excluded keywords with really low numbers from further consideration: *Bactrocera* (2), *Chrysodeixis* (10), *Dichelops* (5), *Gryllus* (2), *Haemagogus* (1), *Heliothis* (9), *Sabethes* (0).

Decision 3: include common names

The main challenge of using common names as keywords is that they can be used in various contexts under different meanings, or they derive from a common word. For example, *esperança* is the common name of bush crickets and is also the word for hope – these orthopterans are named *esperança* because finding them symbolizes good luck, or hope. Searching for this word yielded 570 (+ 33, changing the ç for c) but the first 350 were all “mistakes,” that is, used as hope, and not the insect; and indeed, a single title was within the T&Ds caught with insect orders and generic keywords. The same applied to *efêmera* (23 results, all mistakes) [common name of Ephemeroptera, and the word for ephemerous]; *efemérid** (11 results, all mistakes) [common name of Ephemeroptera, and the word for ephemerality]; and *traça* (489 results, 462 mistakes) [common name of Zygentoma and Lepidoptera, and the word or radical for words like *traçar*, to draw or *traçado*, line or *extração*, extraction]. In all these cases, we found basically mistakes, being unworthy to add them to the keyword list.

Another challenge is that common names are regional, and Brazil is large and diverse enough to have sub-regional common names within regions. We tried to dodge that by exhausting common names we could find on google for each order. Except from the cases above (*esperança*, *efêmera*, *efemérid**, *traça*) and the ones listed in the next paragraph, the common names found in the titles of T&Ds are presented in **Figure S1**. From this figure, we also recognized the need to add common names, even with the potential hassle of curating the “wrong” titles that are also caught by this kind of keywords.

As in decision 1, we excluded keywords with really low numbers from further consideration: *bicho-pau* (0) [Phasmida], *crisopídeo* (3) [Neuroptera], *embiídeo* (0) [Embioptera], *formiga-leão* (0) [Neuroptera], *joaninha* (7) [Coleoptera], *lacrainha* (0) [Dermaptera], *mutuca* (12) [Diptera], *perlário* (0) [Plecoptera], *pernilongo* (1) [Diptera], *piolho* (8) [Phthiraptera], *termita* (3) [Blattodea], *tesourinha* (2) [Dermaptera], *vagalume* (8) [Coleoptera], *varejeira* (9) [Diptera].

As a side remark, in a follow up paper, we will analyze publications of Brazilian entomologists. There, we shall explore with appropriate data the gender gap in the context of insect taxa since, for example, we found that the Entomology keyword with most T&Ds was bee, as a common name (Fig. S1). We recovered more female (649) than male (469) students among these T&Ds, growing expressively after 2003, showing that maybe the gender gap can be reverted in sub-fields of Entomology.

Decision 4: use radicals in specific cases like entom* for Entomology, entomofauna, etc.

Choosing to use part of a word (radical) as the keyword or the full spelling is a balance between precision and specificity *versus* being inclusive and catching “wrong” titles, that is, unrelated to

Entomology. In some cases, like Entomology, entomopathogenic, and entomofauna, using entomo* instead of the three keywords is better since this radical is not a common word, used in other contexts to mean different things. In our case, we used entom* since, for instances, *entomólogo* [entomologist] adds the accentuation and is thus not caught in Excel if we used entomo*.

We chose to use radical in the following cases:

a) entom*, explained above
b) inset*, for *inseto*(*s*) [insect(s)], *inseticida*(*s*) [insecticide(s)], *insetário*(*s*) [insectary/ies]. In this case, the word *insetívoro/a* [insectivore] is also caught and must be manually removed.
c) insect*, for Insecta, insect(s)
d) pulg*, for *pulga*(*s*) [flea(s)], pulgão [aphid], pulgões [aphids]
e) cupin*, for *cupins* [termites] and *cupinzeiro*(*s*) [termite hill(s)]

We checked only for a couple of cases whether the radical indeed catches all variants using the online tool venny (Oliveros 2015), and it does. We also used Venn diagrams to explore potential errors that would be introduced (**Fig. S2**). It is worth noting that, in Excel, the * should not be written.

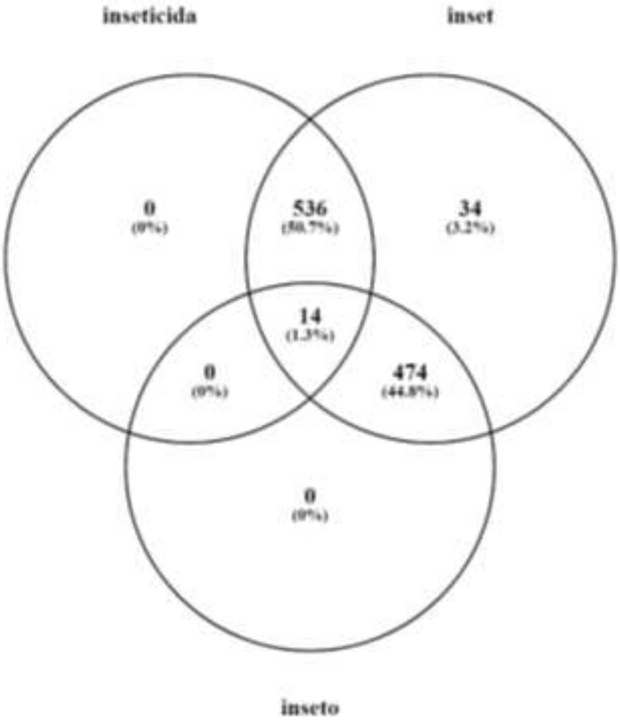

Figure S2: Venn diagrams used to demonstrate that keyword as a radical (here, inset) correctly catches variants (here, *inseticida* [insecticide] and *inseto* [insect]). We also used Venn diagrams to understand if errors were introduced. In this case, the 34 results of “inset” relate to *insetívoro* [insectivore] and *insetário* [insectary].

Decision 5: use scientific names in Latin in their full spelling

We noticed some cases where the insect order was referred to as a vernacular word, like *dipterofauna* or *coleóptero*s. So, we explored for Coleoptera, Diptera, and Lepidoptera if

removing the last vowel (with and without accentuation, for *e.g. coleóptero*) would catch new exclusive results.

By removing the last vowel, we caught additional 0.1-3% D&Ts, except from Dipter* that caught 6% more but 75% of these were due to *Dipteryx*, a plant genus, thus a "mistake." Based on this brief exploration, removing the last vowel did not yield better results.

Another orthographic variant that appeared was the abbreviated form of some orders like Col., Hym., and Lep. We searched these three cases in the abbreviated form but the number of mistakes was in the order of 80% for a subsample of the first 50 D&Ts, and we dropped this possibility without further quantification.

Therefore, in the case of insect orders, the correct spelling is the best strategy. For genus names, we did not see any reason to investigate variants to the correct spelling, so we used any scientific name in Latin in the correct spelling.

Decision 6: use Portuguese words in their correct spelling, including accentuation, hyphenation, or other Latin orthography like cedilla (ç)

The Sucupira database does show some level of punctuation, accentuation, orthography, and other issues – though not so much extra or missing spaces – as any database with more than a million lines, filled for more than 30 years, does. Additionally, Portuguese writing changed, most significantly with the Orthographic Agreement of 1990 (the mandatory transition in Brazil occurred in 2016), so some spelling forms had to be checked. That was more relevant for the common name keywords, in which we focus here, although we also explored variants in cases like *coleoptero*(*s*) and *coleóptero*(*s*) of Decision 5.

For most cases, writing in the correct spelling yielded the best results. For instance, *libélula* [dragonfly] *versus libelula* had respectively 19 and 1 D&Ts, and the 1 caught with libelula (the wrong spelling) was also caught by other keywords. Another example is *afídeo* [aphid], with 35 results, and *afídio* caught 0, *afíd* caught 37 (the extra 2 being mistakes), *afid* caught 12 (all mistakes or caught by other keywords, except from 1 new exclusive result).

After these considerations, we compiled the keyword list including every insect order and the three generic keywords (entom*, inset*, insect*), which yields 9,993 T&Ds. Then we plotted the number of T&Ds gained with additional keywords (**Fig. S1**) and how increments added to the 9,993 above in a cumulative plot (**Fig. S3**). These figures help see where, according to a very inclusive criterium, we stop gaining enough T&Ds. Objectively, the threshold was of contributions of at least 1% (rounded number) of the total exclusive T&Ds found with these additional keywords (5,249). In both **Figures S1 and S3**, that means cutting in *barata* [cockroach].

The final keyword list is:

Archaeognatha OR Auchenorrhyncha OR Blattaria OR Blattodea OR Coleoptera OR Coleorrhyncha OR Collembola OR Dermaptera OR Dictyoptera OR Diplura OR Diptera OR

Embioptera OR Ephemeroptera OR Grylloblattaria OR Grylloblattodea OR Hemiptera OR Heteroptera OR Homoptera OR Hymenoptera OR Isoptera OR Lepidoptera OR Mantodea OR Mantophasmatodea OR Mecoptera OR Megaloptera OR Neuroptera OR Odonata OR Orthoptera OR Phasmatodea OR Phasmida OR Phthiraptera OR Plecoptera OR Protura OR Psocodea OR Psocoptera OR Raphidioptera OR Siphonaptera OR Sternorryncha OR Strepsiptera OR Thysanoptera OR Trichoptera OR Zoraptera OR Zygentoma OR entom* OR insect* OR inset* OR abelha OR Aedes OR formiga OR Apis OR Drosophila OR mosca OR mosquito OR Spodoptera OR Lutzomyia OR borboleta OR vespa OR Anopheles OR Atta OR Bemisia OR cupin* OR Triatoma OR Anticarsia OR besouro OR percevejo OR pulg* OR Culex OR Trichogramma OR Bombyx OR Diaphorina OR Helicoverpa OR cigarrinha OR Anastrepha OR caruncho OR barata.

Data curation

In all databases, we focused especially on columns with people's names, and accentuation mistakes were the most prevalent source of error. We also standardized information for analytical purposes, like date as "month year" and "day/month/year" being changed to "month" and "year" in two columns, or merging "abandonment" and "abandoned." We used two strategies for data cleaning. Firstly, we curated T&Ds caught by the keyword list above using filters in the column Main area for Human and Exact sciences and then each of its Disciplines and read every title.

If a consistent mistake appeared, the second strategy was to filter in the column Title by writing the word in the search engine within the Filter tool in Excel (for example, *Capistrano,* caught because of the keyword *Apis*) and removed all T&Ds found with that filter. We cleaned the database using this strategy for:

- *Insetívor** [insectivore(s)] because of keyword inset*: removed all but those that regarded the diet of insectivore species.
- *Métric** for *cientométrico* [scientometrics], *bibliométrico* [bibliometrics], etc. because of keyword entom*
- *Centomila* because of keyword entom*
- *Vespaziano, Vespasiano* [proper name] because of keyword *_vespa*. A space was inserted before vespa to exclude Bovespa, the Brazilian stock market.
- *Capistrano* [proper name] because of keyword *Apis.*
- *Chácara* [small farm] because of keyword ácar*.
- *Atapulgita* [attapulgite] because of keyword pulg*.
- Double filter in *cigarra* [cicada] and *formiga* [ant] because of the children's tale entitled *A cigarra e a formiga*.

After data curation, the final database of T&Ds caught by keywords related to Entomology sum to 14,448 T&Ds (1% of the total of 1,235,795), with 10,049 of them being theses and 4,399 dissertations, in 1,224 graduate courses (25% of the total of 4.918). We made an exploratory wordcloud (**Fig. S4)** of words in the title of these 14,448 T&Ds (excluding punctuation, words with wrong accentuation, and some prepositions, conjunctions, etc.) using the online tool

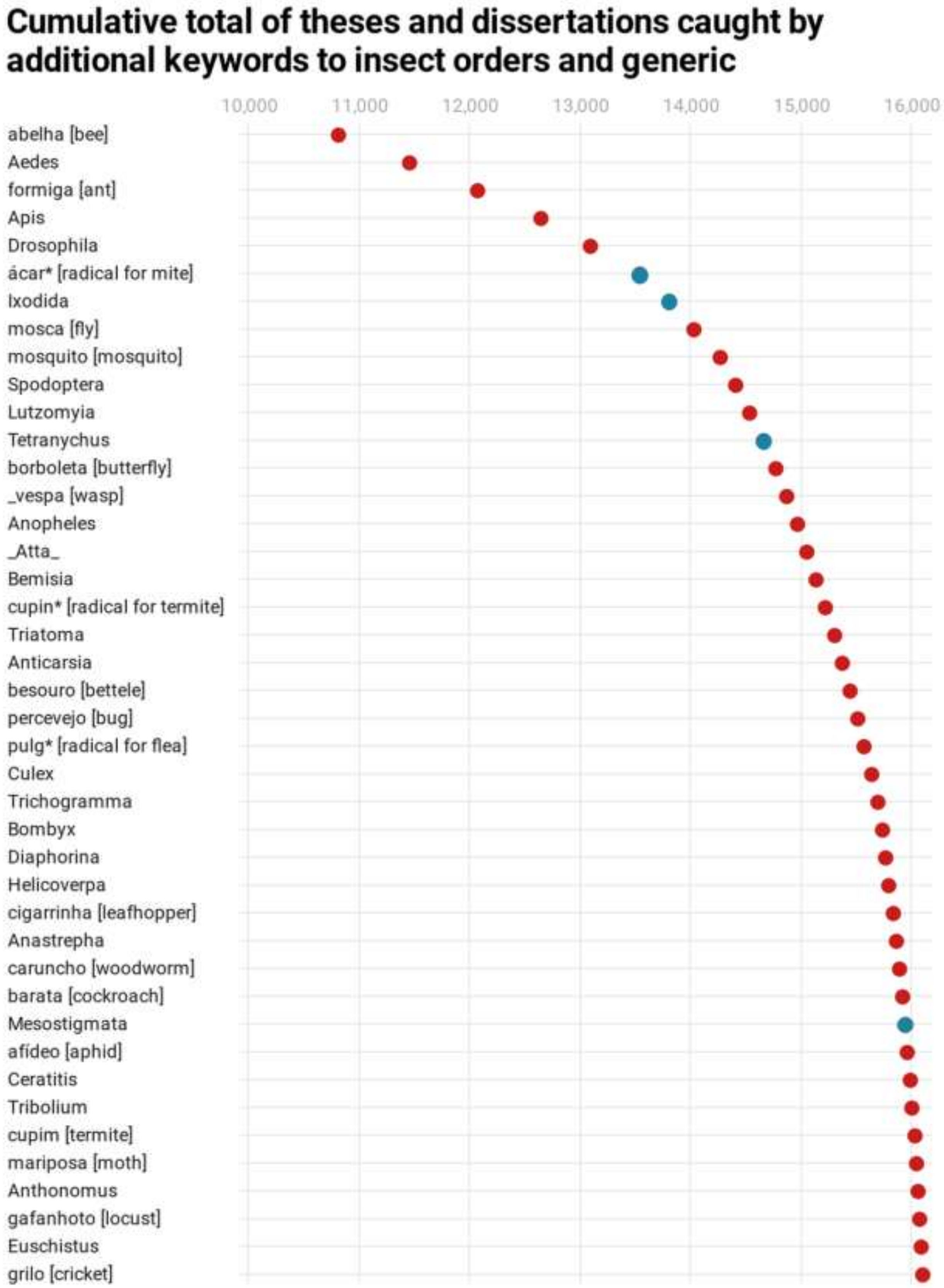

Figure S3: Cumulative plot showing how much additional keywords increase (exclusive T&Ds) to the 9,993 titles caught by insect orders and generic keywords. Keywords are in Portuguese but were translated here in English (in brackets). * indicates radicals, underline indicates a space and, in blue, keywords related to mites (see last section).

WordClouds (https://www.wordclouds.com/). The wordcloud showed the preponderance of studies in Brazil, with the insect order Diptera and dipterans important for basic (*Drosophila*) and medical sciences (*Aedes*), as well as many words related with applied science (like *controle* [(biological) control], *cultura* [culture], *soja* [soya], *milho* [corn], and many words related with bees such as *Apis*, *abelha*, Apidea).

Only 54 graduate courses are responsible for half of these 14,448 T&Ds (**Fig. S5**) and, among these, 9 of 11 graduate courses that formed most masters and doctors are Entomology graduate courses (EGC, **Fig. S5 inset**). These top 11 graduate courses contribute to 25% of the 14,448 T&Ds, and the 9 EGCs are responsible for 22%. The number of T&Ds correlates with the number of professors in each EGC (see Pearson coefficients in the main text), but also with the age of the EGC (Fig. S6), with the earliest EGCs appearing in the 1970's.

Due to the considerable relevance of EGCs in the realm of T&Ds caught by keywords related to Entomology, we decided that only EGCs would be considered for detailed analyses of particular aspects of graduate courses, such as disciplines, gender bias by supervisor/advisor and by student/post-doc, gender bias of coordinators, etc.

In Brazil, there are 12 EGCs. Nine of them are clearly relevant to academic human resources (**Fig. S5 inset**). The remaining three contribute with 51 T&Ds in total, and they are either inactive (UFMS) or too recent (Public Health USP, since 2015). Despite not contributing as much as the other EGCs, we decided to include them, so the detailed analyses of graduate courses done in the main text contemplated all EGCs in Brazil.

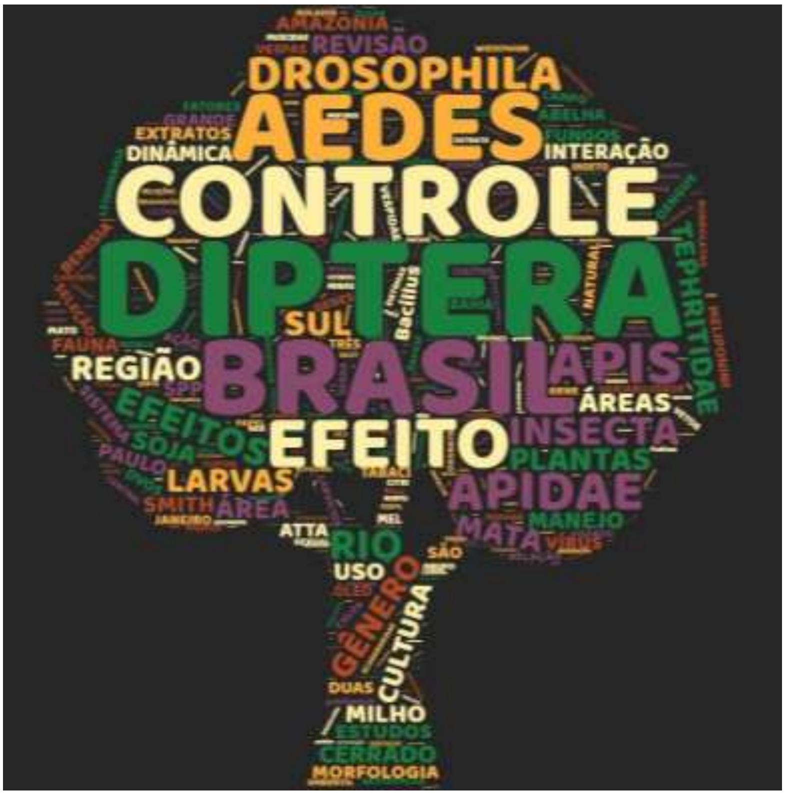

Figure S4: Wordcloud of words in the title of the 14,448 T&Ds caught with the 75 keywords related to Entomology in the CAPES Sucupira platform.

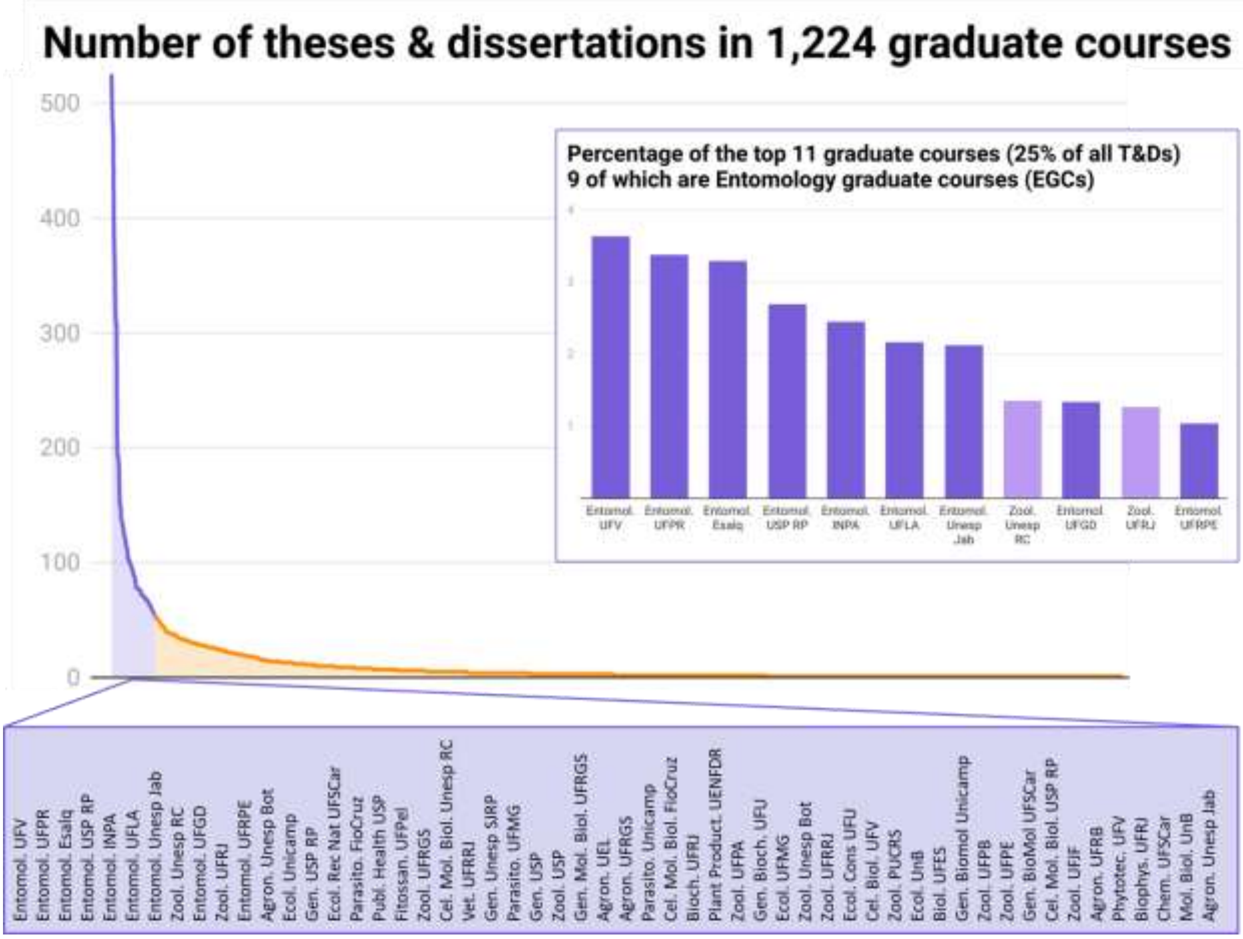

Figure S5: Number of T&Ds caught by keywords related to Entomology, showing in purple the names of those graduate courses responsible for 50% of the total 14,448 T&Ds. The inset histogram focuses on the first 11 graduate courses, that together represent 25% of the total T&Ds, 9 of which are EGCs (dark purple).

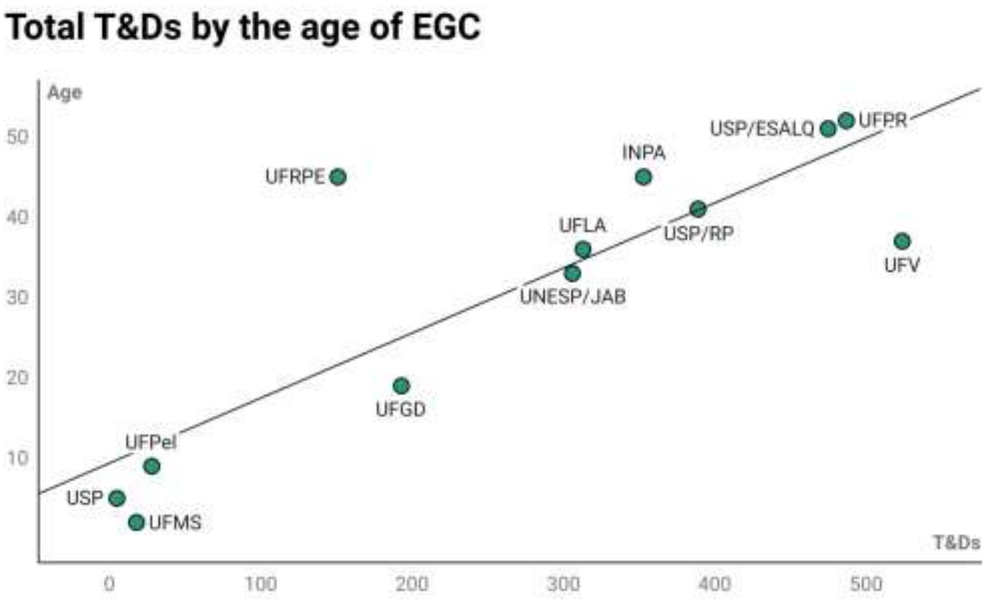

Figure S6: Total number of T&Ds by the age of the EGC. See correlation values in the main text.

Acarology in EGCs

One way to check the adequacy of chosen keywords would be to see the percentage of T&Ds caught in the total universe of T&Ds of these 9 most productive EGCs (**Fig. S5**), with the expectation that nearly 90-100% would be caught with our keywords. The average percentage among these 9 EGCs was 79%, ranging from 68 to 89%, which led us to study the T&Ds our keywords did not catch.

Part of this explanation comes from T&Ds done with mites. Most of the 12 Brazilian EGCs are related to Agronomy, and thus agricultural pest research is a strong research line. Despite mites being arachnids, and not insects, we briefly explored the contribution of mite keywords, looking at six orders (Holothyrida, Ixodida, Mesostigmata, Opilioacarida, Sarcoptiformes, Trombidiformes), two generic keyword (ácar*, acari*), and one genus (*Tetranychus*). We noticed that Acari was a frequently used word in T&D titles and included as a generic keyword (for it also brings *e.g. acaricida*), despite not being an order. This keyword in particular led to too many "mistakes" due to *e.g. sacarina* [saccharrin], *polissacarideo* [polysaccharide], *tabacaria* [tobacconist], *Camacari* [proper name of a town], Peracarida [Crustacea], etc. Using "_acari_" (underline being spaces) caught only 18 results, which is not the real number of Acari in T&D titles – a better search would be, for example, "(Acari_" but this exploration is beyond the scope of our study. In total, we caught 1,725 titles but almost half (736) were mistakes, mostly due to Acari, so we excluded it.

As in decision 1, we excluded keywords with really low numbers from further consideration: Holothyrida (0), Opilioacarida (3), Sarcoptiformes (1), Trombidiformes (10). We plotted the four remaining keywords in blue in **Figures S1 and S3** to illustrate the relative importance of mite research. Within the 9 EGCs, we recalculated the percentage caught with our keywords, including now the four mite keywords, and the average increases to 82%, ranging from 71 to 90%. We thus suggest a dedicated scientometrics and gender bias study to be done for Acarology or, if done for Arachnology, that it considers mite research done within EGCs.

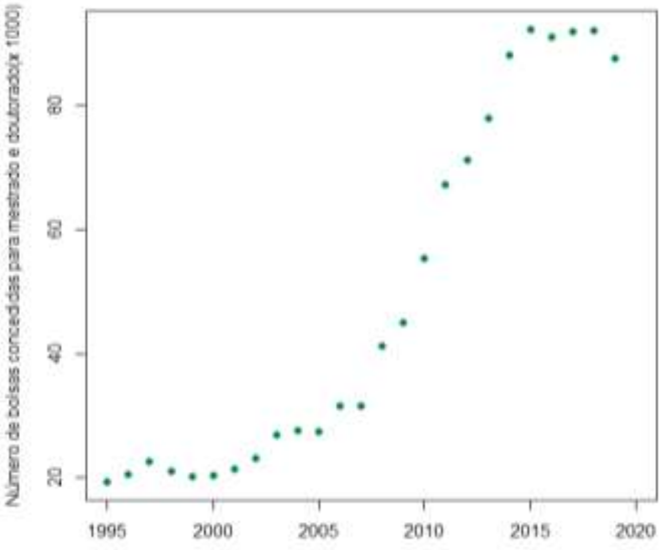

Figure S7: Number of MSc and PhD fellowships granted by CAPES from 1995 to 2019. Source: GEOCAPES (https://geocapes.capes.gov.br/geocapes/).

References

Amabis, J. M. & Martho, G. R. (1990). Biologia Moderna. 1ª edição. Ed. Moderna, São Paulo.

Barua, M., Gurdak, D. J., Ahmed, R. A., & Tamuly, J. (2012). Selecting flagships for invertebrate conservation. *Biodiversity and Conservation*, *21*(6), 1457-1476.

Beutel, R. G., Yavorskaya, M. I., Mashimo, Y., Fukui, M., & Meusemann, K. (2017). The phylogeny of Hexapoda (Arthropoda) and the evolution of megadiversity. In *Proc. Arthropod. Embryol. Soc. Jpn* (Vol. 51, pp. 1-15).

Brusca, R. C. & Brusca, G. J. (2003). Invertebrates. 2nd edition. Sinauer Associates, Sunderland.

Chesters, D. (2019). The phylogeny of insects in the data-driven era. *Systematic Entomology*.

IBGE 2020. https://www.ibge.gov.br/apps/populacao/projecao/index.html?utm_source=portal&utm_medium=popclock&utm_campaign=novo_popclock Accessed Dec 31st 2020

Glänzel, W., Leta, J., & Thijs, B. (2006). Science in Brazil. Part 1: A macro-level comparative study. *Scientometrics*, *67*(1), 67-86.

González-Alcaide, G., Salinas, A., & Ramos, J. M. (2018). Scientometrics analysis of research activity and collaboration patterns in Chagas cardiomyopathy. *PLoS neglected tropical diseases*, *12*(6), e0006602.

Kjer, K. M., Carle, F. L., Litman, J. & Ware, J. (2006). A molecular phylogeny of Hexapoda. *Arthropod Syst Phylogeny*, *64*(1), 35-44.

Kristensen, N. P. (1981). Phylogeny of insect orders. *Annual Review of Entomology*, *26*(1), 135-157.

Leta, J., Thijs, B., & Glänzel, W. (2013). A macro-level study of science in Brazil: seven years later. *Encontros Bibli: revista eletrônica de biblioteconomia e ciência da informação*, *18*(36), 51-66.

Lewinsohn, T. M. & Prado, P.I. (2005). Quantas espécies há no Brasil? *Megadiversidade, 1*, 36-42.

Misof, B., Liu, S., Meusemann, K., Peters, R. S., Donath, A., Mayer, C., ... & Zhou, X. (2014). Phylogenomics resolves the timing and pattern of insect evolution. *Science*, *346*(6210), 763-767.

Oliveros, J. C. 2015. Venny, an interactive tool for comparing lists with Venn's diagrams. https://bioinfogp.cnb.csic.es/tools/venny/index.html

Rafael, J. A., Melo, G. A. R., Carvalho, C. J. B., Casari, S. A., Co, R. (2012). Insetos do Brasil. 1ª edição. Editora: Holos

Storer, T. I. & Usinger, R. L. (1979). Zoologia geral. 5a edição. Companhia Editora Nacional, São Paulo.

Tihelka, E., Giacomelli, M., Huang, D. Y., Pisani, D., Donoghue, P. C., & CAI, C. Y. (2020). Fleas are parasitic scorpionflies. *Palaeoentomology*, *3*(6), 641-653.

Trautwein, M. D., Wiegmann, B. M., Beutel, R., Kjer, K. M., & Yeates, D. K. (2012). Advances in insect phylogeny at the dawn of the postgenomic era. *Annual review of entomology*, *57*(1), 449-468.

Zyoud, S. H. (2016). Dengue research: a bibliometric analysis of worldwide and Arab publications during 1872–2015. *Virology journal*, *13*(1), 1-10.
