# Supplementary material for: The Gender Gap in Brazilian Entomology: an Analysis of the Academic Scenario
Source: Neotrop Entomol. 2021 Nov 12;50(6):859–72. doi: 10.1007/s13744-021-00918-7 (PMC8587496; doi:10.1007/s13744-021-00918-7)
Supplement: Supplementary file 2 — Supplementary file2 (PDF 87 KB) [file 13744_2021_918_MOESM2_ESM.pdf]

Profissionais de insetos no Brasil

Caro(a) profissional,

Você estuda ou trabalha com insetos no Brasil? Você tem na sua linha de pesquisa ou na sua atuação acadêmica ou corporativa a entomologia? Esta é uma pesquisa realizada pela Sociedade Entomológica d[illegible] Brasil (SEB) dentro do Projeto Mulheres na Entomologia, que visa diagnosticar a atuação dos profissionai[illegible] na Entomologia brasileira. Com o projeto será possível retratar a formação e atuação desses profissionai[illegible] no Brasil e a comparação entre gêneros. O projeto é conduzido pela SEB e faz parte do seu processo metodológico a aplicação de questionários, a realização de entrevistas (em grupos e/ou individuais) e o desenvolvimento de pesquisa-ação. Você poderá ser convidado(a) para participar de mais de um deles, caso tenha interesse.

Por favor, leia com atenção as informações e instruções descritas neste documento. No caso de qualque[illegible] dúvida antes, durante ou depois de sua participação, envie-nos um e-mail em secretaria@seb.org.br ou mulheresnaentomo@gmail.com. Caso queira receber os resultados desta pesquisa, enviaremos ao seu email escrito abaixo.

Reiterando a importância da sua participação nesta pesquisa, agradecemos antecipadamente.

Caso conheça outras pessoas atuando com insetos, pedimos que encaminhe o convite para termos a melhor representação do nosso cenário! A sua colaboração neste estudo é MUITO IMPORTANTE, mas a decisão de participar é VOLUNTÁRIA.

Equipe Projeto Mulheres na Entomologia

* Required

1. Email address *

____

2. Você deseja receber os resultados do projeto no seu e-mail?

- [ ] Sim
- [ ] Não

TERMO DE CONSENTIMENTO LIVRE E ESCLARECIDO (TCLE)

Você está sendo convidado(a) a participar de uma pesquisa/survey que está sob a responsabilidade da SEB, sob a diretoria da pesquisadora Dra. Eliane Dias Quintela e coordenação de projeto da Dra. Eliana Maria Gouveia Fontes. Esta pesquisa objetiva diagnosticar a formação e atuação dos profissionais que trabalham com insetos e para tal inicialmente precisamos conhecer as características de sua formação e do contexto no qual você trabalha. O procedimento metodológico desta pesquisa é quantitativo/qualitativo.

Você levará cerca de 10 minutos (tempo previsto) para responder o questionário completo. Não há respostas corretas ou incorretas, então responda cada pergunta conforme a sua opinião/realidade. Os riscos associados a sua participação nessa pesquisa são mínimos, podendo estar associados a estresse, desconforto e tempo de resposta. Os benefícios esperados incluem desde entender características dos(as) profissionais da Entomologia, até fornecer dados para embasar a criação de espaços de discussão da representatividade de gênero, fornecendo elementos para o subsidio de politicas públicas.

Sua participação nessa pesquisa é totalmente voluntária e você tem o direito de decidir não participar e, também, de interromper a sua participação a qualquer momento, por qualquer razão. Se houver algum problema durante o preenchimento online, pedimos que comunique aos contatos disponíveis neste documento.

Nenhum dado pessoal será divulgado sem a sua permissão. Os dados pessoais aqui solicitados têm apenas a finalidade de controle e possível comunicação futura com as pesquisadoras do projeto. Todas as pesquisadoras envolvidas neste estudo garantem o sigilo e a confidencialidade dos dados coletados, os quais serão tratados e apresentados exclusivamente de modo estatístico (que ficarão armazenados de forma segura por, no mínimo, cinco anos).

Caso você tenha qualquer dúvida quanto aos seus direitos como participante de pesquisa, entre em contato com Comitê de Ética em Pesquisa da Pontifícia Universidade Católica do Rio Grande do Sul (CEP-PUCRS) em (51) 33203345, Av. Ipiranga, 6681/prédio 50 sala 703, CEP: 90619-900, Bairro Partenon, Porto Alegre – RS, e-mail: cep@pucrs.br, d[illegible] segunda a sexta-feira das 8h às 12h e das 13h30 às 17h. O Comitê de Ética é um órgão independente constituído de profissionais das diferentes áreas do conhecimento e membros da comunidade. Sua responsabilidade é garantir a proteção dos direitos, a segurança e o bem-estar dos participantes por meio da revisão e da aprovação do estud[illegible] entre outras ações.

Ao dar continuidade a esta pesquisa, você concorda que está ciente das informações apresentadas e que qualquer dúvida, quando manifestada, foi adequadamente esclarecida. Ao concordar com este termo de consentimento, você não abre mão de nenhum direito legal que teria de outra forma. Diante do exposto expresso minha concordância de espontânea vontade em participar deste estudo, autorizando o uso, compartilhamento e publicação dos meus dados e informações de natureza pessoal par[illegible] essa finalidade específica.

3. Se estiver de acordo, manifeste o seu consentimento selecionando a opção “Sim, aceito participar da pesquisa”.

- Sim, aceito participar da pesquisa
- Não

Dados Pessoais

4. Nome completo: *

5. Qual a sua idade?

6. Como você se declara?

- Asiático(a)
- Branco(a)
- Indígena
- Pardo(a)
- Negro(a)
- Outro

7. Qual a sua identidade de gênero? (1) Que se identifica com o sexo que lhe foi designado ao nascer; (2) Possui outra identidade de gênero, diferente da que lhe foi designada ao nascer (3) Não definem sua identidade dentro do sistema binário homem mulher

- Homem cisgênero (1)
- Homem transexual/transgênero (2)
- Mulher cisgênera (1)
- Mulher transexual/transgênera (2)
- Não-binário (3)
- Outro

8. Você possui alguma deficiência?

- Não
- Sim, portador(a) de deficiência auditiva
- Sim, portador(a) de deficiência física
- Sim, portador(a) de deficiência visual
- Outras

9. Você tem filho(s) ou filha(s)?

- Sim
- Não

Filhos(as)

10. Quantos?

____

11. Qual a idade dele(s) ou dela(s)?

____

Formação e Atuação

12. Qual a sua titulação?

- [ ] Técnico
- [ ] Graduação
- [ ] Graduação em andamento (ainda não concluída)
- [ ] Mestrado
- [ ] Mestrado em andamento (ainda não concluída)
- [ ] Doutorado
- [ ] Doutorado em andamento (ainda não concluída)

13. Em que área você obteve sua titulação máxima? ex. Entomologia, Zoologia, Ecologia, Genética...

____

14. Qual o ano de conclusão da titulação máxima?

____

15. Qual a sua principal área de atuação na Entomologia ou aquela que você mais se identific (Ex. Ecologia, Taxonomia, Controle Biológico, etc.)

____

16. Em qual segmento você considera que é sua atuação principal?

- Ensino
- Pesquisa
- Extensão
- Gestão
- Empresa

17. Qual a natureza do seu trabalho principal?

- Público
- Privado

18. Qual o seu local de trabalho principal?

- Instituto Federal de Ensino
- Instituição de pesquisa
- Universidade
- Serviço de extensão rural
- Empresa de consultoria
- Outro

Local de trabalho

19. Qual o seu local de trabalho?

Local de trabalho atual

20. Há quantos anos você trabalha neste local? (marcar 0 para menos de 1 ano)

21. Em qual Unidade da Federação o seu local de trabalho principal se encontra?

- Acre
- Alagoas
- Amapá
- Amazonas
- Bahia
- Ceará
- Distrito Federal
- Espírito Santo
- Goiás
- Maranhão
- Mato Grosso
- Mato Grosso do Sul
- Minas Gerais
- Pará
- Paraíba
- Paraná
- Pernambuco
- Piauí
- Rio de Janeiro
- Rio Grande do Norte
- Rio Grande do Sul
- Rondônia
- Roraima
- Santa Catarina
- São Paulo
- Sergipe
- Tocantins

22. Este é o seu estado de origem?

- Sim
- Não

23. Em qual(is) bioma(s) você atua majoritariamente?

- [ ] Amazônia
- [ ] Caatinga
- [ ] Cerrado
- [ ] Mata Atlântica
- [ ] Pampa
- [ ] Pantanal

24. Qual o seu vínculo no local de trabalho? Marque quantas opções sejam aplicáveis.

- [ ] Autônomo(a) ou prestador(a) de serviço
- [ ] Bolsista
- [ ] Celetista
- [ ] Desempregado(a) no momento
- [ ] Empresário(a) - Tem o seu próprio negócio na área da Entomologia
- [ ] Não atuo na área da Entomologia no meu trabalho principal
- [ ] Servidor(a) público(a)

25. Você atua em algum setor de inovação de empresa pública ou privada (pesquisa, gestão [illegible] outros)?

- Sim
- Não

Setor de inovação de empresa

26. Caso atue em setor de inovação, qual seria?

LinkedIn

27. Você tem LinkedIn?

- Não
- Sim

LinkedIn

28. Qual o seu LinkedIn (URL do seu perfil)?

Área acadêmica

29. Você atua na área acadêmica?

- Sim
- Não

Editoração

30. Você atua na editoração científica?

- Sim
- Não

Editoração científica

31. Qual a sua atuação na editoração científica? (pode marcar mais de uma alternativa)

- [ ] Revisor(a) de artigos
- [ ] Editor(a) associado(a) ou adjunto(a)
- [ ] Membro de comitê ou conselho editorial
- [ ] Editor(a)-chefe

Estamos terminando :) Agradecemos por você ter chegado até aqui!

32. Em seu local de trabalho há mais homens que mulheres?

- ( ) Sim, há mais homens
- ( ) Não, há mais mulheres
- ( ) Homens e mulheres estão em mesma proporção
- ( ) Não se aplica, encontro-me sem emprego no momento

33. Em seu local de trabalho, na sua área de atuação, há mais homens que mulheres em carg[illegible] de liderança?

- ( ) Sim, há mais homens
- ( ) Não, há mais mulheres
- ( ) Homens e mulheres estão em mesma proporção em relação aos cargos de liderança
- ( ) Não se aplica, encontro-me sem emprego no momento

34. Em sua experiência, na sua área de trabalho, há diferenças relacionadas ao gênero (ex. atuação de homens e mulheres)? Por favor explique.

35. Os desafios e oportunidades são iguais para profissionais independentemente do gênerc Marque SIM para concordo e NÃO para discordo. Caso queira complementar a sua respo por escrito, por favor o faça na próxima sessão.

- Sim, concordo
- Não, discordo

36. Agradecemos se quiser complementar a sua resposta da pergunta anterior, relacionada a desafios e oportunidades profissionais relacionados ao gênero.

This content is neither created nor endorsed by Google.

Google Forms
